# Persuasion and gender: experimental evidence from two political campaigns

**DOI:** 10.1007/s11127-024-01192-y

**Published:** 2024-08-05

**Authors:** Vincenzo Galasso, Tommaso Nannicini

**Affiliations:** 1 IGIER, CESIfo & CEPR, Bocconi University, Milan, Italy; 2 IGIER, CEPR & IZA, Bocconi University, Milan, Italy; 3 Department of Social and Political Sciences, Bocconi University, Via Roentgen 1, 20136 Milan, Italy

**Keywords:** Gender differences, Political campaign, Randomized controlled trial, Voting, D72, J16, M37

## Abstract

We investigate differential responses by gender to competitive persuasion in political campaigns. We implemented a survey and a field experiment during two mayoral elections in Italy. Eligible voters were exposed to a positive or negative campaign by an opponent. The survey experiment used on-line videos and slogans. The field experiment used door-to-door canvassing. In both experiments, gender differences emerge. Females vote more for the opponent and less for the incumbent when exposed to positive—as opposed to negative—campaigning. Males do the opposite. These differences cannot be explained by gender identification, ideology, or other voters’ observable attributes.

**Supplementary Information:**

The online version contains supplementary material available at 10.1007/s11127-024-01192-y.

## Introduction

Despite the widespread use of negative campaigning, empirical evidence on its effectiveness in persuading (or demobilizing) voters remains ambiguous (Lau et al., 2007). A substantial body of literature has explored various aspects of negative campaigning, including the closeness of the race, the presence of an incumbent, differences in funding levels, and the characteristics of both politicians and voters, in order to assess its impact. Studies have shown that the effect of negative attacks on voter behavior is contingent upon factors such as the receiver’s tolerance for negativity (Fridkin and Kenney, 2011), their preferences for political candidates (Krupnikov, 2011), their status as core supporters (Glaeser et al., 2005), their levels of hostile or benevolent sexism (Cassese and Holman, 2019), and other individual characteristics. Consequently, the divergent findings reported in the extensive literature on negative campaigning may obscure heterogeneous treatment effects.

A key factor among the personal traits that may influence the impact of various electoral messages is the gender of the receiver.^1^ Indeed, advertisers have traditionally tailored different arguments to convince female and male consumers.^2^ In this paper, we examine the gendered response to negative campaigning. Should we expect male and female voters to react differently as recipients of negative electoral messages from competing politicians? Our theoretical expectations regarding the answer to this question are grounded in the following syllogism.

*Premise one*: Both theory and empirical evidence suggest that women tend to prefer competition and aggressive behavior less than men, also in politics. Fridkin and Kenney (2011, p. 315, Table 2) show that female voters have lower tolerance toward negative advertisements, especially if uncivil or related to personal life. Gilligan (1982) and feminist theory suggest that women prefer less confrontational procedures compared with men; while Ulbig and Funk (1999) theorize that people differ in their “conflict avoidance” attitudes and people who are more likely to dislike conflict are less likely to participate in politics. Outside the realm of politics, females and males are also recognized to differ in their attitudes toward competition and negotiation (see Croson and Gneezy (2009), Bertrand (2010), Niederle and Vesterlund (2011), for reviews).^3^

*Premise two*: Empirical evidence suggests that political candidates attacking their competitor(s) may experience a backlash effect, as voters evaluate them less along the valence dimension and believe they are less cooperative (Carraro and Castelli, 2010; Lau and Rovner, 2009; Galasso et al., 2023).^4^ In particular, experimental evidence in the lab shows that negative campaigning (as opposed to positive) increases the voters’ belief that the attacker is competitive, rather than cooperative, and that he/she might not be a good elected official (Galasso et al., 2023).

*Conclusion*: Female voters should evaluate negative campaign messages less favorably than male voters. This is exactly the theoretical hypothesis that we test. To do that, we implemented a survey experiment in the field and a large scale field experiment during two electoral campaigns in Italy, and analyzed the differential effect of negative vs. positive electoral campaigning on turnout and voting behavior of male and female voters.

First, we ran a survey experiment during the 2011 electoral race for mayor in Milan, which featured a female incumbent facing a male main opponent. We randomized several items of the opponent’s electoral campaign—videos, letters, slogans—in a negative vs. positive tone. We departed from existing studies on negative vs. positive campaigning by administering a “complete” electoral campaign, exploiting the notion that different communication tools could potentially reinforce each other (e.g., see Green and Gerber (2004)).^5^ The “in the field” component of our experimental design comes from collecting turnout and voting choices through a final survey, run in the days immediately after the election. For this election, we were also able to exploit an “unexpected event during survey design” in the spirit of Muñoz et al. (2020), in order to further examine the effect of an attack by the (female) incumbent to the (male) main opponent on voters’ perceptions.

Second, we conducted a large-scale field experiment during the 2015 mayoral electoral race in Cava de’ Tirreni, a midsize town in the South of Italy, featuring a male incumbent and male opponents. In this experiment, our randomized treatments consisted of negative versus positive canvassing. This involved door-to-door campaigning by volunteers who aimed to engage in personal interactions with eligible voters. Volunteers knocked on apartment doors and distributed electoral materials, either directly to the voters or in their mailboxes. Canvassing occurred in the three weeks leading up to the election. Volunteers were instructed to leave either negative (against the incumbent) or positive electoral materials and to use a coherent script when engaging with voters, according to a pre-determined randomization protocol. Subsequently, we conducted a post-electoral survey among a sample of eligible voters in both treated and control groups to gather information on turnout and actual votes, which were the main outcomes of interest.

Overall, we thus use different methodologies—survey, natural, and field experiment—in different geographic environments (the largest city in the North of Italy and a midsize city in the South), with different gender races—mixed in Milan and “all males” in Cava de’ Tirreni—and exploit several electoral campaign instruments (video ad, slogan, flyer, and canvassing). All this experimental evidence points in the same direction. The gender of the receiver matters: Positive campaign is more effective than negative among women, while the opposite happens for men. Going negative backfires with a female audience, while it proves effective with a male audience. These results have implications for the public choice literature as they suggest that besides appealing to voters’ perceived self-interest, politicians may also exploit voters’ preferences for campaigning style.^6^

This paper contributes to a growing literature on gender differences by providing experimental findings on a differential response (by gender) to political persuasion strategies—whether negative or positive. Non-experimental studies have addressed the possible gender difference in the behavioral response to the different tone of electoral campaigns: Goldstein and Freedman (2002) exploit National Electoral Study data and report no gender difference in the effect of campaign attacks on electoral participation; using survey and observational data, Kahn and Kenney (2011) show instead that females are less tolerant than males to (both civil and uncivil) negative messages. Cassese and Holman (2018) show that female candidates are more vulnerable to attacks targeting personal traits stereotypically associated with women, at least when such attacks originate from a male politician (as their experimental design holds the gender of the attacking candidate constant). Teele et al. (2018) demonstrate that voters penalize politicians who fail to conform to stereotypical family roles, which places female candidates at a disadvantage due to the disproportionate amount of time women spend on family responsibilities compared to men. Cassese and Holman (2019) study the gendered dimension of negative campaigning by looking at reactions to Donald Trump attacking Hillary Clinton in 2016 for playing the “woman’s card.” They experimentally find that, once exposed to the attack, hostile sexists increased their support for Trump, while benevolent sexists increased their support for Clinton. These effects are larger than those related to gender or other attributes of voters. Alexander et al. (2020) reveal gender differences in voter reactions to corruption scandals involving their favored political party.

Our empirical findings also contribute to a large literature on the effects of negative campaigning on electoral turnout and (individual) voting behavior.^7^ In their seminal paper, Ansolabehere et al. (1994) exposed a sample of Californian eligible voters to a single (negative vs. positive) political ad, aired during a commercial break. Using responses from a post-test questionnaire, they found that the negative ad reduced average voting intentions by 5%. Arceneaux and Nickerson (2010) implemented a field experiment, in which volunteers personally delivered a political message to their treatment groups to find that, while canvassing is effective in influencing voters, there is little evidence of a differential effect between negative and positive ads.^8^ Other studies on negative campaigning used aggregate and survey data and classified the negativity of the actual campaign advertisement. Most of these papers find either no impact of negative campaigning (Wattenberg and Brians, 1999), or even supporting evidence for a “stimulation” effect on electoral turnout (Finkel and Geer, 1998; Freedman and Goldstein, 1999; Kahn and Kenney, 1999; Goldstein and Freedman, 2002; Clinton and Lapinski, 2004; Brooks and Geer, 2007). A meta-analytic assessment of this literature by Lau et al. (2007) reports inconclusive results: Negative campaigns are neither effective to win votes, although they may be more memorable, nor seem to depress turnout. Our results suggest that the lack of an average treatment effect may mask large gender effects.

## The Milan experiment

### Survey experiment

We examine the effects of positive vs. negative campaigning on a sample of (male and female) eligible voters, who accepted to participate in a series of online surveys prior to the election for mayor of Milan in May 2011. The two main candidates were Letizia Moratti, the female incumbent supported by a center-right coalition, and Giuliano Pisapia, the main (male) opponent supported by a center-left coalition. A Milan-based commercial survey company (“CE &Co”) was contacted to run the online surveys. They used different techniques (such as exploiting their existing online panel or producing new contacts using phone books) to construct an initial sample of about 1536 eligible voters, aged between 18 and 65. The sample was stratified along three dimensions: (i) neighborhood, (ii) age group, and (iii) gender. As common in survey experiments, the sample was not representative of the electorate aged from 18 to 65 years in the 2011 Milan election, since it is difficult to convince certain demographic groups to participate to online surveys.^9^ The internal validity of the experiment, however, is guaranteed by the randomization protocol.

**Fig. 1:**
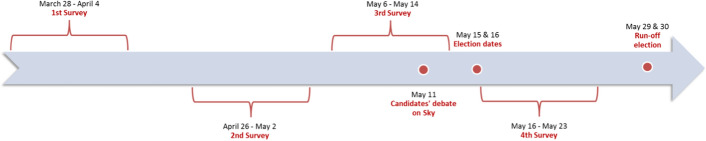
Timing of the Experimental Design in Milan. *Notes* All dates refer to 2011. The timeline reports the starting and ending dates of the four online surveys; the date of the candidates’ debate on Sky TV; and the dates of the elections (first round and runoff). The first (pre-randomization) survey profiled the eligible voters in the sample. The second survey administered the first two informational treatments: video interviews with the candidates; campaign slogans. The third survey administered the last two informational treatments: open letters to voters; video ads endorsed by the candidates. The fourth (post-election) survey elicited voting behaviors

Our survey experiment was implemented between March and May 2011 by providing four surveys to the eligible voters in our online sample (see Fig. 1). The first survey was administrated with the goal of obtaining relevant personal information (gender, age, marital status, education) and specific information on political and social attitudes (political orientation, voting behavior in previous local and national elections, exposure to the media, knowledge of local politics). Respondents to the initial survey were then randomly assigned to our three (treatment and control) groups. The second survey, only for individuals in the treatment groups, contained the first wave of the electoral campaign: a video interview and a campaign slogan. The third survey, again to the treatment groups only, contained the second wave of the electoral campaign: an open letter to the voters and a video ad. The mayoral election took place on May 15 and 16. The (fourth and last) survey was conducted for all three groups immediately after the election. This survey collected information on self-reported electoral outcomes (such as turnout and actual vote for the candidates) and personal perceptions about the electoral campaign.^10^ These answers provided the “in the field” component of the experiment, and are the main outcomes of the empirical analysis.

Not all 1536 individuals profiled in the first survey responded also to the subsequent surveys. In our analysis, we use the 1,140 voters who participated to the fourth survey on electoral outcomes and declared whether they voted or not. The main characteristics of the estimation sample are summarized at Table A.1 in Appendix A, which provides descriptive statistics by treatment group. Besides standard demographic characteristics and education, we measure the ideological position of each voter, the interest in politics, and the knowledge about local politics (“did not know mayor” meaning that the name of the incumbent mayor was misreported). All variables but the nonresponse dummy (“missing”) come from the first survey, which provided the (pre-randomization) individual characteristics. The first column reports the “missing” dummy for the original sample of 1536 individuals profiled in the first survey; the dummy is equal to one if the (profiled) individual did not answer to the fourth survey and therefore does not belong to the final (estimation) sample.

The estimation sample is largely composed of females (59%), college graduates (44%), and married individuals (48%). There is a large share of individuals younger than 30 (23%), and only very few respondents have a low interest in politics (4%) or did not know the name of the mayor (3%). Table A.2 in Appendix A shows that all of these observable characteristics are balanced across treatment groups, with the only exception of the information measure at the 10% significance level. The attrition rate caused by no responses to the fourth survey (something that we could not check ex ante) is also balanced across groups. This confirms the (ex post) validity of the experimental design. Additionally, all observable characteristics are perfectly balanced by treatment status also within gender strata. Tables A.3 and A.4 in Appendix A show that observable covariates are balanced across treatment groups for both females and males, respectively. Most importantly, the nonresponse rate—which is determined after our treatments took place—is also balanced across treatment groups by gender. We also replicated standard randomization checks within gender strata (see Table A.5). These checks confirm the validity of the randomization within gender. As a result, the randomization outcome allows us to estimate the causal impact of positive vs. negative campaigning for both men and women separately.

Unlike the existing experimental literature, in this survey experiment, we exposed individuals in the treatment groups to an entire electoral campaign by the opponent, composed of four electoral tools either with a positive or a negative tone. All individuals in the two treatment groups were also exposed to the same electoral campaign by the incumbent, again characterized by the same four electoral tools. For each tool, we randomized whether survey respondents were first exposed to the opponent’s or to the incumbent’s campaign message. Providing an entire electoral campaign increases the strength, and the realism, of our treatment, but at the price of reducing the possibility of pinning down the effect of each campaign tool. The informational treatments coexisted with the real campaign, going on independently of our surveys, and therefore their effects (if any) operated at the margin. However, we designed the experiment so that the intensity of the overall treatment could be strong, as different campaign items with the same tone might reinforce each other (see Green and Gerber (2004)), especially on individuals who did not want or did not have time to follow the real campaign closely.

The first tool of the opponent’s randomized campaign was a 100-second video interview to the candidate sitting at his office desk, given in a positive or negative tone on the same issues. The second tool was the opponent’s main campaign slogan: respectively “Pisapia for Mayor = Less Traffic & More Green. A Change for Milan is Possible” in the positive tone campaign, and “5 Years of Moratti = More Traffic & Less Green. A Change for Milan is Possible” in the negative one (see Figures A.1 and A.2 in Appendix A). The third tool was a letter to the voters. In the positive tone, the letter described the opponent’s main projects for the future of Milan; in the negative tone, the letter charged the incumbent for her mistakes while in office. The final tool was a 60-second video ad endorsed by the opponent on relevant issues for the city (transportation, pollution, Expo). Each video ad addressed the same issues, with the same format and in the same setting, and was proposed in either a positive or a negative tone. To evaluate whether the tone of our campaign was correctly perceived by the individuals in our treatment groups, the forth survey contained questions which allowed for an ex-post analysis. In Appendix A, we provide further details on these informational treatments and the English translation of all texts.^11^

To measure the causal impact of positive vs. negative campaigning by gender we estimate the following linear probability model by OLS:

1 $$\begin{aligned} Y_{i}=\alpha _{1}POS_{i}+\alpha _{2}NEG_{i}+\beta _{1}POS_{i}\times FEMALE_{i}+\beta _{2}NEG_{i}\times FEMALE_{i}+\delta FEMALE_{i}+\varepsilon _{i}, \end{aligned}$$

where *POS* and *NEG* are dummies that identify the exposure to positive or negative campaign, respectively, *FEMALE* is a dummy identifying female voters, and standard errors are clustered by ZIP code to account for spatial correlation.

The outcome variables of interests are first-round self-declared actual electoral choices and immediate reactions to the treatments. In particular, we focus on self-declared turnout, vote for the incumbent, the opponent and the other (minor) candidates in the first round; and on agreement with the treatment, and trust in the candidates.

Since we want to estimate the differential impact that positive and negative campaigning may have by gender, we choose to present our results showing separately in our figures the treatment effect of positive vs. negative campaign for males (corresponding to the null $\alpha _{1}-\alpha _{2}=0$ in the previous equation) and for females (corresponding to the null $(\alpha _{1}+\beta _{1})-(\alpha _{2}+\beta _{2})=0$). We also report the differential treatment effect of positive vs. negative campaign between males and females, corresponding to the null $\beta _{1}-\beta _{2}=0$. In our tables, of course, we report all estimated coefficients and relevant hypothesis tests.

**Fig. 2:**
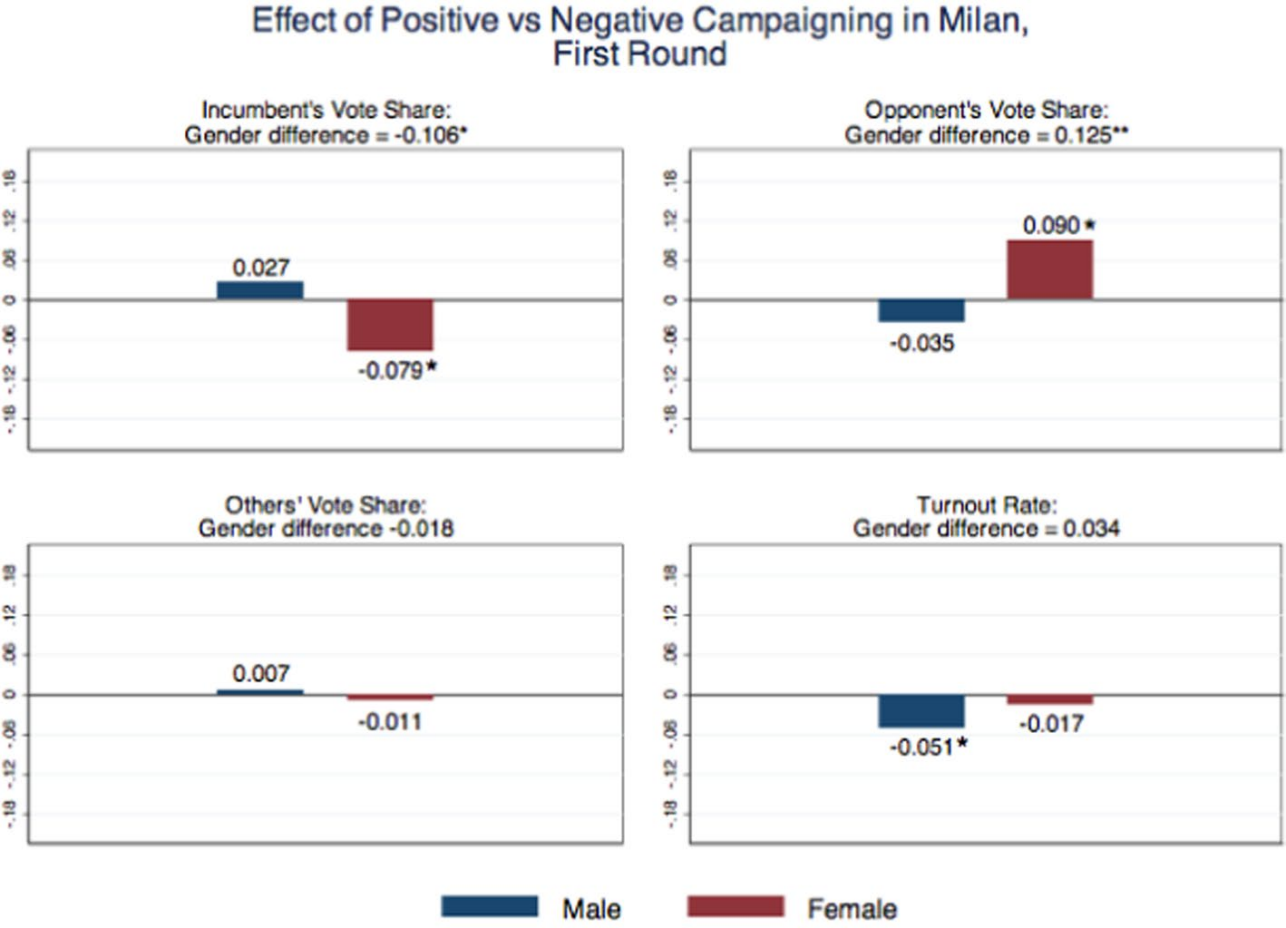
Effect of positive vs. negative campaigning in Milan, first round. *Notes* The “male” estimate captures the treatment effect of positive vs. negative campaign for males: $\alpha _1-\alpha _2$ in Eq. (1). The “female” estimate captures the treatment effect of positive vs. negative campaign for females $(\alpha _1+\beta _1)-(\alpha _2+\beta _2)$. The “gender difference” estimate captures the differential treatment effect of positive vs. negative campaign between males and females: $\beta _1-\beta 2$. Significance at the 10% level is represented by *, at the 5% level by **, and at the 1% level by ***

Figure 2 shows the results on voting choices. As opposed to positive, negative campaigning increases male turnout by 5 percentage points (at the 10% significance level), but no difference emerges by gender. Gender differences are pronounced when we look at the candidates’ vote shares. Females vote more for the opponent (by 9 percentage points) and less for the incumbent (by 8 points) when they are exposed to the opponent’s positive campaign, as opposed to the negative one. The effect for males is of the opposite sign, but not statistically significant. As a result, gender differences are there, as the differential treatment effect of negative vs. positive campaign by gender is large both for the opponent vote share, 12.5 percentage points (statistically significant at 5% level) and for the incumbent vote share, 10.6 percentage points (statistically significant at 10% level). There are no significant effects on the cumulative vote shares of the other (minor) candidates. Table 1 provides all estimated coefficients of Eq. (1) and also shows hypothesis testing with respect to the control group.

**Table 1:** Effects of Campaign Information by Gender in Milan

	Turnout rate	Opponent’s vote share	Incumbent’s vote share	Others’ vote share
Positive campaign ($\alpha _1$)	0.031	$-$0.110*	0.127**	$-$0.018
	[0.043]	[0.059]	[0.054]	[0.063]
Negative campaign ($\alpha _2$)	0.082**	$-$0.075	0.100	$-$0.025
	[0.037]	[0.069]	[0.061]	[0.054]
Positive campaign $\times$ Female ($\beta _1$)	$-$0.080	0.190**	$-$0.207***	0.018
	[0.051]	[0.080]	[0.075]	[0.070]
Negative campaign $\times$ Female ($\beta _2$)	$-$0.114**	0.065	$-$0.101	0.036
	[0.049]	[0.083]	[0.077]	[0.065]
Female	0.061	0.004	0.067	$-$0.071
	[0.040]	[0.071]	[0.057]	[0.052]
*P*-value H1: $\alpha _1 + \beta _1$=0	0.068*	0.154	0.119	0.994
*P*-value H2: $\alpha _2 + \beta _2$*=0*	0.289	0.851	0.982	0.770
*P*-value H3: $\alpha _1 - \alpha _2$*=0*	0.092*	0.435	0.619	0.876
*P*-value H4: $\alpha _1 + \beta _1 - (\alpha _2 + \beta _2)=0$	0.556	0.062*	0.074*	0.776
*P*-value H5: $\beta _1 - \beta _2=0$	0.365	0.035**	0.076*	0.785
*P*-value H6: $\alpha _1 + \alpha _2 = 0$	0.137	0.132	0.033**	0.694
*P*-value H7: $\alpha _1 + \beta _1 + \alpha _2 + \beta _2=0$	0.102	0.460	0.342	0.870
*P*-value H8: $\beta _1 + \beta _2=0$	0.043**	0.104	0.034**	0.656
Obs	1,140	912	912	912

Estimated OLS regression: $Y_i=\alpha _1 POS_i+\alpha _2 NEG_i + \beta _1 POS_i\times FEMALE_i + \beta _2 NEG_i\times FEMALE_i +\delta FEMALE_i +\varepsilon _i$. (H1) Treatment effect of positive vs. no campaign for females: $\alpha _1 + \beta _1 = 0$. (H2) Treatment effect of negative vs. no campaign for females: $\alpha _2 + \beta _2 = 0$. (H3) Treatment effect of positive vs. negative campaign for males: $\alpha _1-\alpha _2=0$. (H4) Treatment effect of positive vs. negative campaign for females: $(\alpha _1+\beta _1)-(\alpha _2+\beta _2)=0$. (H5) Differential treatment effect of positive vs. negative campaign between males and females: $\beta _1-\beta _2=0$. (H6) Treatment effect of any campaign vs. no campaign for males: $\alpha _1+\alpha _2=0$. (H7) Treatment effect of any campaign vs. no campaign for females: $(\alpha _1+\beta _1)+(\alpha _2+\beta _2)=0$. (H8) Differential treatment effect of any campaign vs. no campaign between males and females: $\beta _1+\beta _2=0$. Robust standard errors are in brackets. Significance at the 10% level is represented by *, at the 5% level by **, and at the 1% level by ***

Also the immediate reactions to the tools of the electoral campaign show that male voters agree less with the incumbent messages (video and slogan) and trust the incumbent less when they are exposed to the opponents’ negative campaign messages, as opposed to the positive ones (see Figures A.3 and A.4 and Table A.6 in Appendix A). The opposite occurs for female voters, so that the differential treatment effect of negative vs. positive campaign across gender is again large, particularly for the perceptions about the incumbent.

What drives these different behaviors? Answers to a question in the third survey allow us to exclude that they are due to different perceptions, by gender, about the tone of the campaign. We then use information obtained in the first survey to test whether they may depend on other aspects that are recognized in the literature to differ by gender, such as education, political ideology (Edlund and Pande, 2002), preferences toward competition (Bertrand, 2010) or cooperation (Niederle, 2016), and preferences for public policy (Cavalcanti and Tavares, 2011; Funk and Gathmann, 2013). Table A.7 in Appendix A shows in fact that, in our sample, female respondents differ along several observable characteristics, such as age, marital status, left-wing orientation, and interest in politics.^12^ The results from adding to our baseline specification, one at a time, each of the variables that differ by gender—young, college, left and low interest in politics—and their interaction with the treatment indicator (see Table A.8 in Appendix A) show that the introduction of these additional explanatory variables (and of the respective interaction terms) does not eliminate (or even reduce) the differential gender effect of positive vs. negative campaign (corresponding to the null $\beta _{1}-\beta _{2}=0$ in Eq. 1). These observable channels cannot explain our results.

### Unexpected event design

Since the 2011 Milan election featured a mixed-gender race, between a female incumbent and a male opponent, gender identification may drive our results (see Akerlof and Kranton (2000)). Females may dislike negative advertising against a female candidate, whereas males may accept (or even like) the male opponent attacking the female incumbent. Would the results be different if a female politician attacked a male politician? The emergence of an unexpected event during the 2011 Milan election allows us to test this situation, since the female incumbent staged an aggressive campaign attack against the male opponent in a TV debate. On May 11, during a political debate broadcast on Sky TV, Ms. Moratti accused Mr. Pisapia of taking part in a car robbery with other communist terrorists in his youth. Exploiting the rules of the debate, Ms. Moratti used her closing statement for her attack, so that Mr. Pisapia was unable to reply and defend himself. The opponent was clearly shocked by the attack, and refused to shake hands with the incumbent at the end of the TV show. Only after the debate, Mr. Pisapia was able to explain to the press that he had been fully and immediately acquitted from the charge, and announced his intention (not carried out) to sue Ms. Moratti. The negative attack had a huge echo in local and national news media, and marked a turning point in the campaign.^13^

Thanks to the (exogenous) timing of our survey experiment, we can exploit this episode as an “unexpected event during survey design” in the spirit of Muñoz et al. (2020), in order to study the effect of negative campaigning by a female politician (the incumbent) against a male politician (the opponent). In fact, our third survey was still under way when the Sky TV show was aired (see Fig. 1). As a result, some individuals had already participated in the third survey, while others (14% of the sample) had not. We exploit the timing of the survey response, in order to evaluate the impact of a negative attack carried out by a female candidate against a male candidate. To implement this evaluation, we must restrict the analysis to the outcomes measured in the third survey, because at the time of the fourth all voters had already come to know about TV episode.

To further examine this event, we also acquired Twitter data related to the 2011 Milan election from a London-based social media monitoring platform company (“FACE”). We then performed a sentiment analysis to assess the effects of the Sky TV debate. We obtained an initial dataset of around 87,000 tweets regarding the 2011 Milan electoral race for mayor, covering a period of two months (from April 1 to May 31). We considered tweets for which we could obtain information about the gender of the user, and that contained the word “Moratti” and/or “Pisapia.”Furthermore, to avoid potential assignment-bias in the sentiment analysis, we excluded the tweets that contain the name of both the incumbent and the opponent. In the end, we are left with almost 45,000 tweets referring only either to “Pisapia” or to “Moratti,”sent from accounts for which we can recognize the gender of the sender. On these tweets, we perform a sentiment analysis to study the effect of the negative campaign episode. In particular, using the gender of the sender, we test whether Ms. Moratti’s attack during the TV debate had a differential gender effect on the tone (negative or positive) of the tweets just before vs. just after the Sky TV show.

To perform our sentiment analysis, we initially identified a list of stems (roots of a word or of many words), which are relevant to infer the sentiment towards a candidate. A positive stem is related to an emotion, such as joy or love, or to an expression of political support, such as “vote for.”Conversely, a negative stem is related to a pessimistic emotion, or to an expression of political dislike. We also included some emoticons as they are widely used on Twitter to express feelings. The complete list contains 108 stems, of which 54 are coded as positive and 54 as negative (see Table B.1 in Appendix B). For each tweet, we thus count the number of “negative”and “positive” words, and we construct four indicators. The Moratti (Pisapia) Negative Index measures the difference between negative and positive words in a tweet that refers only to Moratti (Pisapia). The Moratti (Pisapia) Negative Dummy indicates whether there are more negative than positive words in a tweet referring only to Moratti (Pisapia).

To test the differential gender effect of Ms. Moratti attack during the Sky TV debate, we use two specifications for both our data sources. First, we estimate the following OLS model:

2 $$\begin{aligned} Y_{i}=\alpha _{1}AFTER_{i}+\beta _{1}AFTER_{i}\times FEMALE_{i}+\delta FEMALE_{i}+\varepsilon _{i}, \end{aligned}$$

where $Y_{i}$ is either the response to the survey or the tone of the tweets, as captured by the indicators described above, and the dummy $AFTER_{i}$ captures respectively whether the individual did the survey or sent the tweet after the Sky TV show or before.

**Fig. 3:**
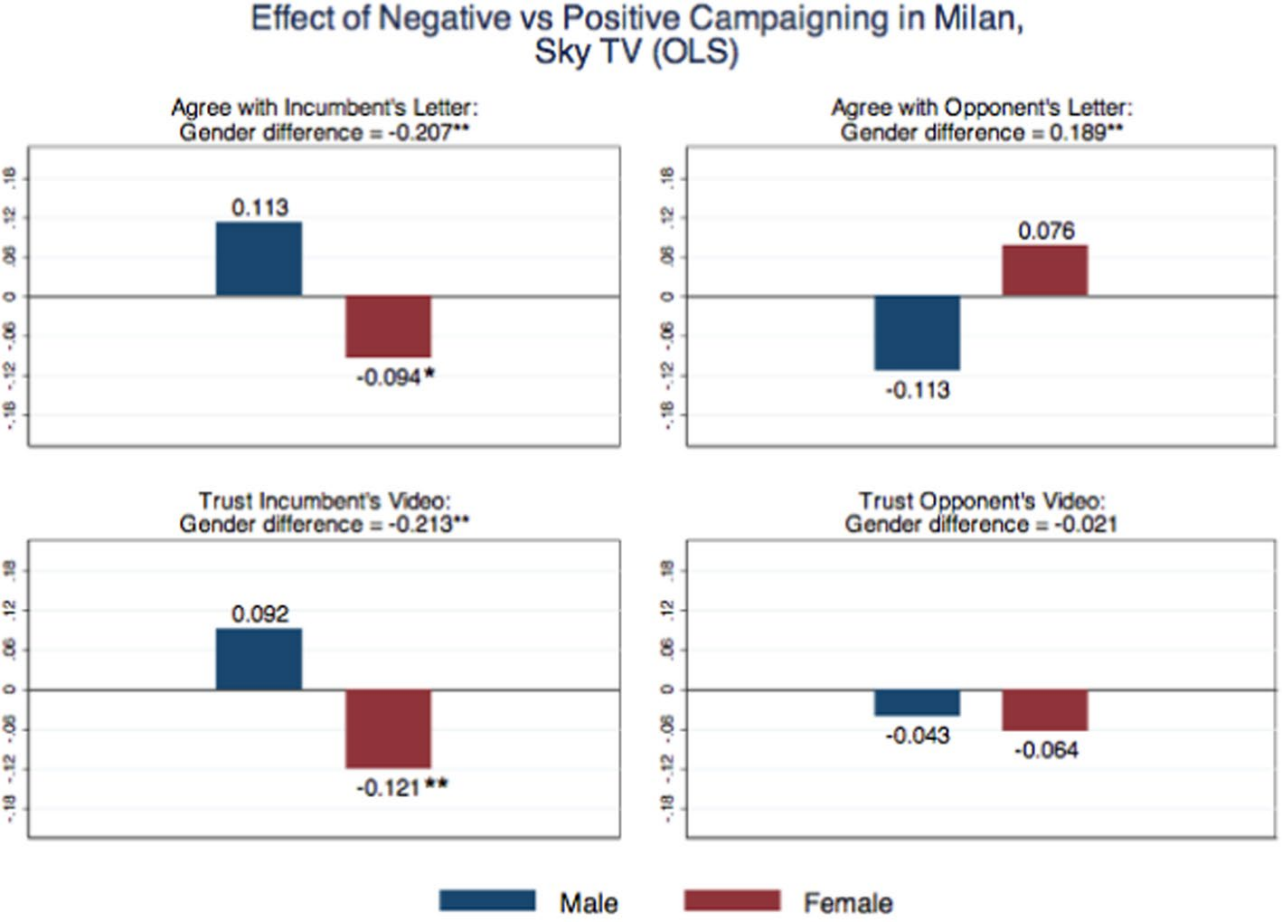
Effect of negative vs. positive campaigning in Milan, Sky TV (OLS). *Notes* The “male” estimate captures the treatment effect of positive vs. negative campaign for males: $\alpha _1-\alpha _2$ in Eq. (1). The “female” estimate captures the treatment effect of positive vs. negative campaign for females $(\alpha _1+\beta _1)-(\alpha _2+\beta _2)$. The “gender difference” estimate captures the differential treatment effect of positive vs. negative campaign between males and females: $\beta _1-\beta 2$. Significance at the 10% level is represented by *, at the 5% level by **, and at the 1% level by ***

We are aware that individuals doing the survey or sending tweets before or after the TV show may be different along some unobservable dimension. This is why, in our second specification, we augment (2) with a spline third-order polynomial in the distance from the time of the event:

3 $$\begin{aligned} Y_{i}=\alpha _{1}AFTER_{i}+\beta _{1}AFTER_{i}\times FEMALE_{i}+\delta FEMALE_{i}+f(DISTANCE_{i})+\varepsilon _{i}, \end{aligned}$$

where $DISTANCE_{i}$ is measured in minutes. This amounts to a regression discontinuity (RD) design in the distance from the Sky TV show. We also restrict the analysis to tweets sent 24 h before and 24 h after the broadcast of the show. When using data from our third survey, we consider the same outcomes analyzed in Figure A.4 and in Table A.6 in Appendix A, and estimate whether female and male voters who replied to the survey after the Sky TV show have different evaluations on the quality of both the incumbent’s and the opponent’s messages. Clearly, these are intention-to-treat effects, because we are unable to know whether those individuals who replied after the show actually heard about the TVep isode. Results are reported in Fig. 3 and in Table 2 (OLS specification in Panel A and RD specification in Panel B).

Also in this case, female voters tend to punish the candidate who went negative, even though this time it is a woman; they agree less with the letter and trust less the video by the incumbent after the TV attack. On the contrary, male voters do not punish the female incumbent. If anything, they tend to rally in her favor even if she went negative against a male candidate. As a result, the differential effect of the negative attack by gender is large for the perceptions about the incumbent. Table 2 also shows that the OLS and the RD specifications provide very similar findings.

Using the four indicators previously described for the sentiment of the tweets, we estimate whether female and male voters tweeting after the Sky TV show modified the tone of the tweets toward the two candidates. These represent intention-to-treat effects, as we are unable to assess whether those who sent tweets after the show actually knew about the episode. Results are reported in Figure B.1 and in Table B.2 in Appendix B (OLS specification in Panel A and RD specification in Panel B). The number of negative tweets against Ms. Moratti increased significantly (at 1% level) after the Sky debate among females, while remaining constant among males. The difference across gender is large and statistically significant at 5% level. The number of negative tweets against Mr. Pisapia —and their intensity, as measured by the net number of negative words— increased significantly (at 1% level) among males, not among females. Table B.1 in Appendix B shows that again the OLS and the RD specifications provide very similar findings.

**Table 2:** Effects of Sky TV Show on 3$^{rd}$ Survey Outcomes

	Agree with opponent’s letter	Agree with incumbent’s letter	Trust opponent’s video	Trust incumbent’s video
*Panel A. OLS specifications*				
After Sky ($\alpha _1$)	$-$0.113	0.113	$-$0.043	0.092
	[0.069]	[0.078]	[0.086]	[0.079]
After Sky $\times$ Female ($\beta _1$)	0.189**	$-$0.207**	$-$0.021	$-$0.213**
	[0.087]	[0.079]	[0.109]	[0.083]
Female	$-$0.024	$-$0.045	0.067	$-$0.033
	[0.042]	[0.038]	[0.040]	[0.034]
*P*-value H1: $\alpha _1 + \beta _1$=0	0.166	0.091*	0.273	0.041**
*Panel B. RD specifications*				
After Sky ($\alpha _1$)	$-$0.142	0.139	$-$0.121	0.071
	[0.089]	[0.088]	[0.117]	[0.093]
After Sky $\times$ Female ($\beta _1$)	0.182**	$-$0.201**	$-$0.040	$-$0.219**
	[0.086]	[0.077]	[0.109]	[0.083]
Female	$-$0.021	$-$0.050	0.069*	$-$0.041
	[0.043]	[0.039]	[0.041]	[0.035]
*P*-value H1: $\alpha _1 + \beta _1$=0	0.631	0.409	0.113	0.116
Obs	762	762	762	762

Panel A reports the OLS specifications: $Y_i=\alpha _1 AFTER_i+ \beta _1 AFTER_i\times FEMALE_i + \delta FEMALE_i +\varepsilon _i$; where $AFTER_i$ is a dummy equal to one if the voter responded before the Sky TV show was aired, and equal to zero otherwise. Panel B reports the RD specifications: $Y_i=\alpha _1 AFTER_i+ \beta _1 AFTER_i\times FEMALE_i + \delta FEMALE_i +f(DISTANCE_i)+\varepsilon _i$; where *f*(.) is a spline third-order polynomial control function, and $DISTANCE_i$ is the distance from the time of the show measured in minutes. (H1) Treatment effect of positive vs. negative campaign for females: $\alpha _1+\beta _1=0$. Robust standard errors are in brackets. Significance at the 10% level is represented by *, at the 5% level by **, and at the 1% level by ***

This unexpected event design, which leverages the attack by the female incumbent against the male main opponent during a TV show, although it may have lower internal validity compared to the other experimental setups, validates the findings of our survey experiment in Milan. Female voters exhibit a dislike for negative campaigning, and this holds true even when the attack originates from a female candidate targeting a male candidate.

## The field experiment in Cava de’ Tirreni

Our field experiment was run during the 2015 municipal election in Cava de’ Tirreni, a midsize town (around 46 thousand eligible voters) in the South of Italy. The main candidates were the incumbent mayor, Marco Galdi, from the center-right coalition, Vincenzo Servalli, the candidate from the center-left coalition, and Armando Lamberti, a candidate supported by three civic lists. We randomized the canvassing component of the Lamberti campaign, administering two information treatments: a positive campaign about his ideas for the future of the city and a negative campaign about the mistakes of the incumbent (Mr. Galdi) in the previous term. Hence, the negative (vs. positive) electoral campaign consists of a male candidate attacking another male candidate in an all-men race. This allows us to evaluate the robustness of our gender results to different patterns of gender identification of voters with candidates. Moreover, exploiting a field experiment and in a different political context also improves the external validity of our research findings.

The experiment was implemented between April 16 and June 12, 2015. A first phone survey was conducted by “IPR Feedback,” a Salerno-based commercial survey company, between April 16 and April 30. “IPR feedback” used the Cava de’ Tirreni public phone database to obtain an initial random sample of about 1,400 eligible voters. The first survey was administrated with the goal of obtaining relevant personal information (gender, age, marital status, education, number of children), as well as more specific information on political and social attitudes (political orientation, voting behavior in the previous local election, individual perceptions about the ideological positioning of the mayoral candidates, individual opinion regarding the more pressing problem that the mayor should address). Additionally, the respondents were asked some questions to elicit their degree of competitiveness (self-reported and stemming from actual participation to sport competitions or other contests), and their view on the importance of cooperative behavior in life.^14^

Voters were then treated with positive or negative canvassing (or not treated) by volunteers of the Lamberti campaign, who attempted to reach voters by knocking on their doors and buzzing their intercoms (see Figures C.1 and C.2 in Appendix C). Elections took place on May 31 and a follow-up post-electoral survey was conducted immediately after to collect information on self-reported outcomes (turnout and actual vote), voters’ perceptions about the candidates’ ideology, and about the tone of their electoral campaign. Not all individuals participated in both surveys. Therefore, our final (estimation) sample consists of 857 voters, who answered to the second survey.

The main characteristics of this sample are summarized at Table C.1 in Appendix C, which provides descriptive statistics by treatment group. Besides standard demographic characteristics and education, we measure the ideological position of each voter, and the preferences for competition and cooperation. The estimation sample is largely composed of females (74%), and married individuals (76%) with children (83%). The share of individuals younger than 30 years (7%), left wing supporters (13%), college graduate (22%) and who participated in a competition or contest (19%) is limited. The first column of Table C.1 reports that 55% and 51% of the surveyed eligible voters, who belong respectively to the positive and negative treatment group, were not personally reached by the volunteers.^15^ Additionally, all observable characteristics are perfectly balanced across treatment status also within gender strata. Tables C.2 and C.3 in Appendix C show that observable covariates are balanced across treatment groups for both females and males, respectively. As in the Milan experiment, we replicated randomization checks within gender strata (see Table C.4 in Appendix C). These checks confirm the validity of the randomization outcome within gender.

Our treatments consisted of positive and negative messages administrated through door-to-door canvassing and the delivery of electoral materials to mailboxes. During the three weeks prior to the election, a campaign team of twenty young volunteers, supporters of Mr. Lamberti, knocked on doors of private residences, and buzzed private residences’ intercoms, to engage in personal interactions with eligible voters. Electoral materials were also left in the mailboxes of the those eligible voters who were not engaged in personal interactions. As a result of this experimental design, we thus have two different campaigning formats (or intensities): *strong*, made of canvassing with flyers and hangers, and *weak*, made of flyers and hangers only.^16^

In this field experiment, we randomized at the electoral precinct level. Cava has 55 electoral precincts, which were randomly assigned to tree groups: positive treatment (18 precincts, with 15,424 eligible voters), negative treatment (18 precincts with 15,424 eligible voters), and control group (19 precincts with 15,174 eligible voters), which did not receive any treatment. Table C.5 in Appendix C reports the ex ante balance tests of predetermined variables at the precinct level. The available variables refer to the previous elections for mayor in Cava de’ Tirreni in 2006 and 2010. For both elections, they include the number of eligible voters (absolute and by gender), the voter share of the center-right, center-left and other candidates, as well as the voter share of the different party lists.^17^ For all of these variables, our precinct-level randomization is perfectly balanced.

While being largely exploited in the US, as part of “get out the vote” strategies, canvassing represented a novelty for Italian politics.^18^ We approached Mr. Lamberti and proposed him to run an experiment using canvassing as an electoral campaign tool. He accepted, and decided to launch a campaign called “Around the city listening to Citizens.” The volunteers were provided by the candidate, and they underwent a one-day training stage with the authors and the field manager.

Volunteers did either positive canvassing, by emphasizing Mr. Lamberti’s ideas, or negative canvassing, by concentrating on the incumbent (Mr. Galdi) wrong-doing while in office. We randomized our negative vs. positive treatments by means of canvassing (which included both personal interaction and electoral materials) or electoral materials left in the mailboxes. The electoral materials consisted of a flyer and a hanger. All these tools were designed by professionals under our direction and in collaboration with the Lamberti campaign. The look of the positive and negative version of flyers and hangers (colour, portray of the candidate, symbols of the civic lists) is identical (see Figures C.3 to C.6 in Appendix C). Also the topic and even the length of the different slogans were the same. The positive flyer emphasized the benefits of the “next five years with Lamberti” while the negative flyer emphasized the failures of the “last five years with Galdi.” Analogously, volunteers were provided with a similar script in the positive and negative treatment to approach the voters (see Appendix C for further details on these informational treatments and the English translation of all texts). Of course, the material distributed to the voters, and the discussion that followed when the volunteers were able to gain personal access to them, differed depending on the tone (negative or positive) of the treatment.^19^

After the election, we used the post-electoral survey to repeat these tests on our respondents. For each of the three main candidates (Galdi, Servalli and Lamberti), respondents were asked to define the political position of the candidate and to assess whether he ran a positive or negative campaign. Survey answers clearly indicates that the tone of the campaign was correctly perceived, both by male and female voters. Table C.6 in Appendix C shows that the positive vs. negative treatment has no effect on the beliefs about the ideological positions of the main candidates (falsification test). The treatments have also no effect on the perceptions about the tone of the campaign by the incumbent and by the other (non-experimental) candidate. The only sizable impact—by almost 27 percentage points—is on the perception that the (experimental) opponent ran a negative campaign against the incumbent, and there are no gender differences in this perception.

**Fig. 4:**
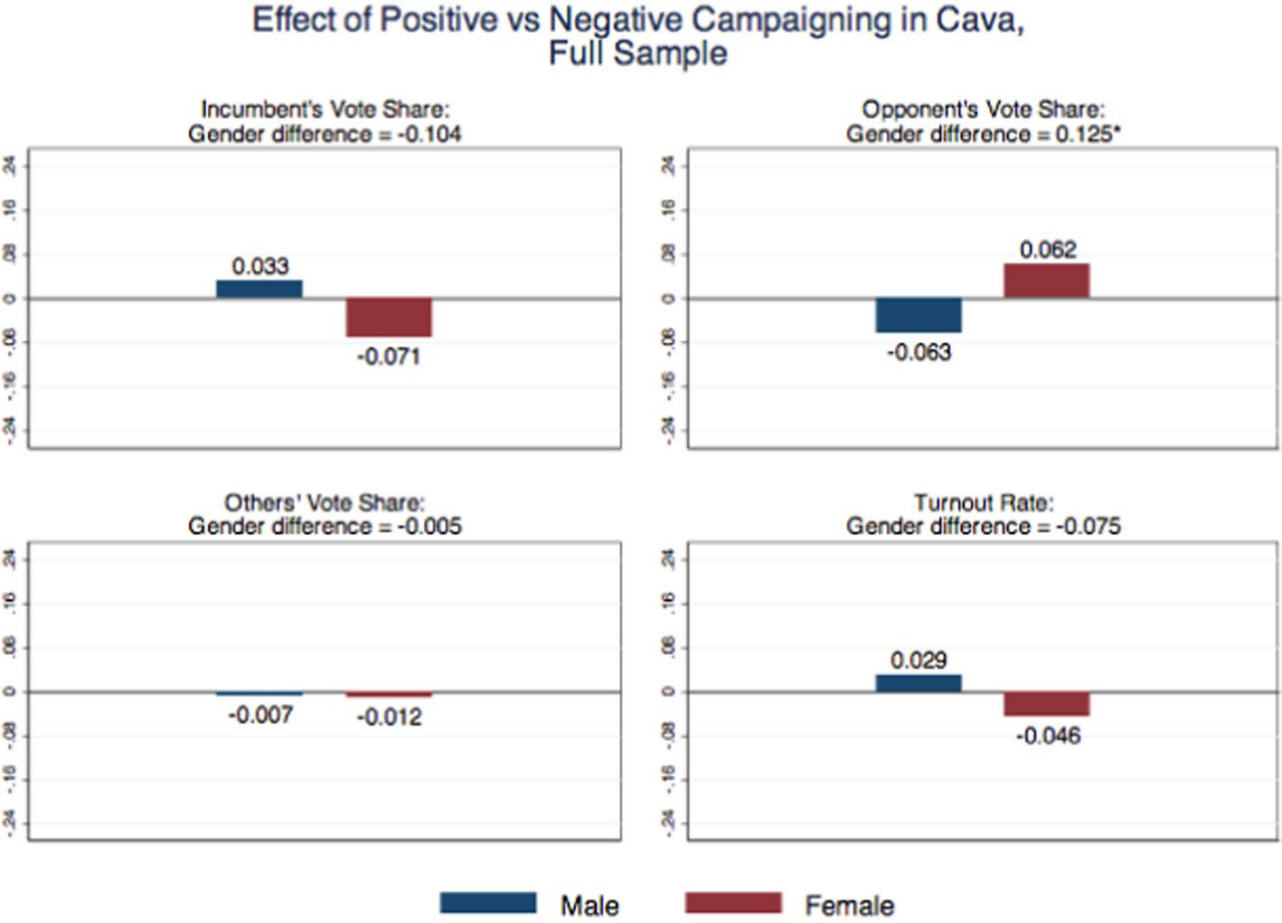
Effect of positive vs. negative campaigning in Cava, full sample.*Notes* The “male” estimate captures the treatment effect of positive vs. negative campaign for males: $\alpha _1-\alpha _2$ in Eq. (1). The “female” estimate captures the treatment effect of positive vs. negative campaign for females $(\alpha _1+\beta _1)-(\alpha _2+\beta _2)$. The “gender difference” estimate captures the differential treatment effect of positive vs. negative campaign between males and females: $\beta _1-\beta 2$. Significance at the 10% level is represented by *, at the 5% level by **, and at the 1% level by ***

Clearly, the informational treatments coexisted with the overall campaign, and therefore their effects (if any) operated at the margin. However, our canvassing was the only door-to-door campaigning done in Cava either by Mr. Lamberti or by the other candidates. Typical tools in the campaign were press releases, candidates’ interviews with local media (newspapers and TV channels), candidates’ speeches at local events, street posters and flyers.

**Table 3:** Effects of Campaign Information by Gender in Cava, Full Sample

	Turnout rate	Opponent’s vote share	Incumbent’s vote share	Others’ vote share
Positive campaign ($\alpha _1$)	0.098*	0.026	$-$0.151	0.099
	[0.059]	[0.045]	[0.101]	[0.109]
Negative campaign ($\alpha _2$)	0.069	0.089	$-$0.184*	0.106
	[0.060]	[0.056]	[0.099]	[0.109]
Positive campaign $\times$ Female ($\beta _1$)	$-$0.134**	0.052	0.009	$-$0.062
	[0.068]	[0.064]	[0.118]	[0.129]
Negative campaign $\times$ Female ($\beta _2$)	$-$0.059	$-$0.073	0.113	$-$0.057
	[0.068]	[0.068]	[0.118]	[0.129]
Female	0.060	0.050	$-$0.001	$-$0.080
	[0.053]	[0.040]	[0.096]	[0.100]
*P*-value H1: $\alpha _1 + \beta _1 = 0$	0.300	0.082*	0.019*	0.598
*P*-value H2: $\alpha _2 + \beta _2 = 0$	0.747	0.683	0.266	0.469
*P*-value H3: $\alpha _1 - \alpha _2 = 0$	0.563	0.271	0.670	0.933
*P*-value H4: $\alpha _1 + \beta _1 - (\alpha _2+\beta _2) = 0$	0.188	0.183	0.230	0.852
*P*-value H5: $\beta _1 - \beta _2 = 0$	0.218	0.089*	0.281	0.963
*P*-value H6:$\alpha _1 + \alpha _2 = 0$	0.121	0.170	0.071*	0.300
*P*-value H7: $\alpha _1 + \beta _1 + \alpha _2 + \beta _2 = 0$	0.657	0.178	0.051*	0.468
*P*-value H8: $\beta _1 + \beta _2 = 0$	0.115	0.853	0.569	0.606
Obs	857	448	448	448

Estimated OLS regression: $Y_i=\alpha _1 POS_i+\alpha _2 NEG_i + \beta _1 POS_i\times FEMALE_i + \beta _2 NEG_i\times FEMALE_i +\delta FEMALE_i +\varepsilon _i$. (H1) Treatment effect of positive vs. no campaign for females: $\alpha _1 + \beta _1 = 0$. (H2) Treatment effect of negative vs. no campaign for females: $\alpha _2 + \beta _2 = 0$. (H3) Treatment effect of positive vs. negative campaign for males: $\alpha _1-\alpha _2=0$. (H4) Treatment effect of positive vs. negative campaign for females: $(\alpha _1+\beta _1)-(\alpha _2+\beta _2)=0$. (H5) Differential treatment effect of positive vs. negative campaign between males and females: $\beta _1-\beta _2=0$. (H6) Treatment effect of any campaign vs. no campaign for males: $\alpha _1+\alpha _2=0$. (H7) Treatment effect of any campaign vs. no campaign for females: $(\alpha _1+\beta _1)+(\alpha _2+\beta _2)=0$. (H8) Differential treatment effect of any campaign vs. no campaign between males and females: $\beta _1+\beta _2=0$. Robust standard errors are in brackets. Significance at the 10% level is represented by *, at the 5% level by **, and at the 1% level by ***

To investigate how females and males react to negative communication, in an electoral race featuring a male incumbent and all male opponents, we estimate Eq. (1) by OLS. Our variables of interest, collected in the second survey, are the (self-reported) voting outcomes: turnout at election, vote for the incumbent, for the opponent who did canvassing, or for the others. Figure 4 and Table 3 show the results on voting choices for the weak treatment, which corresponds to the full sample of voters, i.e., voters in the control group and voters in the treatment groups, regardless of whether personally reached by the canvassing. Negative campaigning (as opposed to positive) increases the votes for the opponent among men and reduces them among women. Although neither effect is statistically significant, the gender difference is large (12.5 percentage points) and statistically significant at 10% level.

**Fig. 5:**
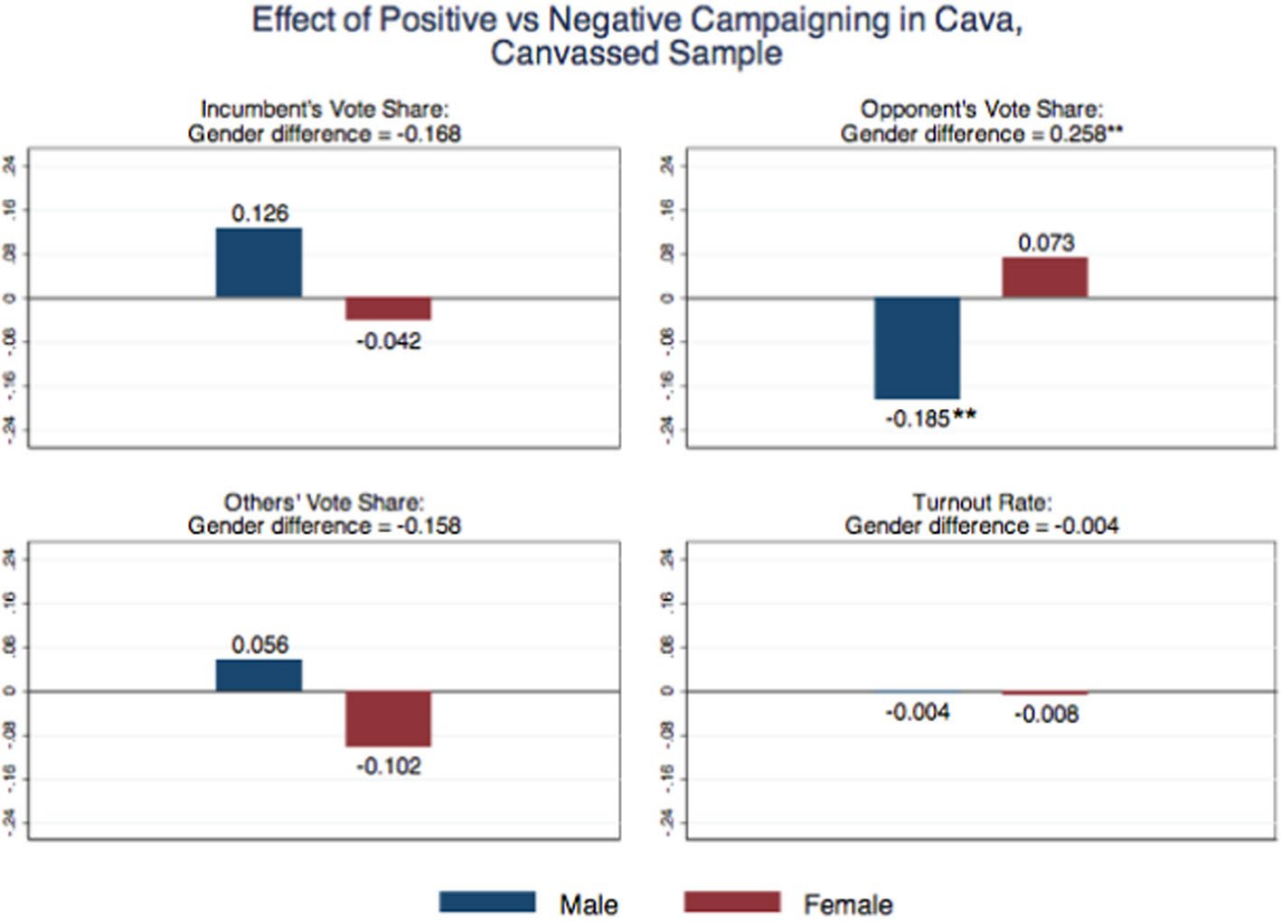
Effect of positive vs. negative campaigning in Cava, canvassed sample. *Notes* The “male” estimate captures the treatment effect of positive vs. negative campaign for males: $\alpha _1-\alpha _2$ in Eq. (1). The “female” estimate captures the treatment effect of positive vs. negative campaign for females $(\alpha _1+\beta _1)-(\alpha _2+\beta _2)$. The “gender difference” estimate captures the differential treatment effect of positive vs. negative campaign between males and females: $\beta _1-\beta 2$. Significance at the 10% level is represented by *, at the 5% level by **, and at the 1% level by ***

Figure 5 displays the results for the strong treatment, which corresponds to the sample of canvassed voters, namely those individuals who were personally reached by our volunteers, either by intercom or at their apartments, and of voters in the control group. In this case, negative campaigning (as opposed to positive) has a strong positive (18.5 points) and statistically significant (at 5% level) effect on the opponent vote share among male voters. The reduction among female voters remains of similar magnitude as before (and not statistically significant). Hence, the gender difference is large (25.8 points) and statistically significant at 5% level. A negative, albeit not statistically significant, effect emerges on the incumbent vote share from negative campaigning among male voters. Table 4 provides the detailed estimation results for the strong treatment.

**Table 4:** Effects of Campaign Information by Gender in Cava, Canvassed Sample

	Turnout rate	Opponent’s vote share	Incumbent’s vote share	Others’ vote share
Positive campaign ($\alpha _1$)	0.041	$-$0.031	$-$0.144	0.166
	[0.074]	[0.031]	[0.117]	[0.123]
Negative campaign ($\alpha _2$)	0.045	0.154*	$-$0.270***	0.110
	[0.070]	[0.082]	[0.099]	[0.125]
Positive campaign $\times$ Female ($\beta _1$)	$-$0.063	0.159**	$-$0.036	$-$0.122
	[0.087]	[0.075]	[0.138]	[0.153]
Negative campaign $\times$ Female ($\beta _2$)	$-$0.059	$-$0.099	0.132	0.036
	[0.083]	[0.100]	[0.125]	[0.152]
Female	0.060	0.050	$-$0.001	$-$0.080
	[0.053]	[0.041]	[0.096]	[0.100]
*P*-value H1: $\alpha _1 + \beta _1 = 0$	0.628	0.060*	0.014**	0.622
*P*-value H2: $\alpha _2 + \beta _2 = 0$	0.757	0.345	0.073*	0.094*
*P*-value H3: $\alpha _1 - \alpha _2 = 0$	0.956	0.015**	0.189	0.649
*P*-value H4: $\alpha _1 + \beta _1 - (\alpha _2+\beta _2) = 0$	0.875	0.372	0.618	0.338
*P*-value H5: $\beta _1 - \beta _2 = 0$	0.963	0.021**	0.188	0.334
*P*-value H6: $\alpha _1 + \alpha _2 = 0$	0.480	0.211	0.035**	0.199
*P*-value H7: $\alpha _1 + \beta _1 + \alpha _2 + \beta _2 = 0$	0.615	0.059*	0.011**	1.183
*P*-value H8: $\beta _1 + \beta _2 = 0$	0.389	0.659	0.677	0.739
Obs	560	282	282	282

Estimated OLS regression: $Y_i=\alpha _1 POS_i+\alpha _2 NEG_i + \beta _1 POS_i\times FEMALE_i + \beta _2 NEG_i\times FEMALE_i +\delta FEMALE_i +\varepsilon _i$. (H1) Treatment effect of positive vs. no campaign for females: $\alpha _1 + \beta _1 = 0$. (H2) Treatment effect of negative vs. no campaign for females: $\alpha _2 + \beta _2 = 0$. (H3) Treatment effect of positive vs. negative campaign for males: $\alpha _1-\alpha _2=0$. (H4) Treatment effect of positive vs. negative campaign for females: $(\alpha _1+\beta _1)-(\alpha _2+\beta _2)=0$. (H5) Differential treatment effect of positive vs. negative campaign between males and females: $\beta _1-\beta _2=0$. (H6) Treatment effect of any campaign vs. no campaign for males: $\alpha _1+\alpha _2=0$. (H7) Treatment effect of any campaign vs. no campaign for females: $(\alpha _1+\beta _1)+(\alpha _2+\beta _2)=0$. (H8) Differential treatment effect of any campaign vs. no campaign between males and females: $\beta _1+\beta _2=0$. Robust standard errors are in brackets. Significance at the 10% level is represented by *, at the 5% level by **, and at the 1% level by ***

Gender differences thus emerge also in this all-men race, with a male opponent attacking a male incumbent. As shown in Fig. 5, these differences are mainly due to male voters, who reward the negative campaigning of the (male) opponent against the (male) incumbent. Female voters have more moderate (not statistically significant) reactions, which however go in the opposite direction. What drives these gender differences? Exploiting questions in our post-electoral survey, in which voters were asked whether different candidates run positive or negative campaigns, we can rule out the fact that male and female voters had different perceptions about the tone of the electoral campaign. Gender differences in the behavioral response to political campaigning may be due to observable differences between males and females along other dimensions. Table C.7 in Appendix C shows in fact that, in our sample, respondents differ by gender along several observable characteristics, including their preferences for competition and cooperation.^20^ We test for these possible channels in Table C.8 in Appendix C, which shows results for the effects of the negative and positive treatments on the opponent’s vote share and the incumbent’s vote share, when to our baseline specification we add, one at a time, each of the following variables and its interaction with the treatment indicator: young, college, left, and preferences for competition and cooperation. These observable channels do not explain our results.

## Conclusion

Our experimental evidence from two electoral campaigns in Italy strongly suggests that the gender of voters receiving negative (vs. positive) messages does indeed matter. In our randomized campaigns, a positive (vs. negative) electoral campaign by the opponent increased his vote share and reduced the incumbent’s vote share among female voters. Conversely, the opposite effect occurred among male voters, who rallied in favor of the politician sending negative ads. Although our results may be somewhat underpowered and we did not pre-register the main gender hypothesis, we find it reassuring that they are robust to different environments (size and location of the city), gender composition of the race (mixed and all male), experimental methodologies (survey, event, field), and electoral campaign instruments (videos, slogans, flyers, canvassing).

Our findings contribute to the literature on gender differences. In addition to other well-recognized disparities in political ideology, risk aversion, preferences for competition, or public policy, we show that gender differences also exist in the behavioral response to political persuasion. Our empirical tests suggest that this difference is not driven by gender identification, as it emerges in three different scenarios: a man attacking a woman, a woman attacking a man, and a man attacking a man. It is therefore striking that in all of these scenarios, females evaluate negative campaign messages less favorably than males, corroborating our theoretical prior that negativity makes politicians perceived as less cooperative, and this is particularly penalized by female voters. Finally, as our findings extend from the North to the South of Italy, which are characterized by very different gender norms, we believe that their external validity extends beyond Italian politics.

## Supplementary Information

Below is the link to the electronic supplementary material.

Supplementary file 1 (pdf 12016 KB)

## Footnotes

1 Gender has also been extensively studied as a politician’s attribute, examining its impact on public policy (Chattopadhyay and Duflo, 2004), party selection (Bagues and Esteve-Volart, 2012; Baltrunaite et al., 2014), and government duration (Gagliarducci and Paserman, 2012).

2 Research on consumer behavior suggests that advertisements linking the advertised product to outperforming others positively influence males’ purchase intentions (Prakash, 1992). Refer to Kahn and Kenney (2011) and Preece and Stoddard (2015) for studies on political messages considering the gender of the receiver.

3 For instance, women tend to shy away from competition, and their performance worsens if there’s conflict (Niederle and Vesterlund, 2011). A neuropsychological literature shows that women have a lower reactivity than men to stressful situations, such as watching a scary or violent movie (Baron-Cohen et al., 2005).

4 The backlash is intensified when a candidate initiates a negative campaign rather than responding to a prior attack (Peterson and Djupe, 2005). Moreover, female candidates are more prone to experiencing a backlash when engaging in negative campaigning (Herrnson et al., 2003).

5 Previous experiments on negative campaigning have typically randomized a single campaign tool, such as a video (Ansolabehere et al., 1994) or canvassing (Arceneaux and Nickerson, 2010).

6 Stratmann (2006) shows that campaign spending is important in increasing the candidates’ vote share and Zakharov (2009) analyzes the interaction between campaign spending and political polarization.

7 More generally, the effectiveness of electoral campaigns in mature democracies is the subject of a large literature, including, among others, Ansolabehere and Iyengar (1995), Gerber and Green (2000), Green and Gerber (2004), Gerber et al. (2003), Nickerson (2008), Dewan et al. (2014), Huber et al. (2022). Typically, these studies rely on either small scale experiments for partisan ads, or on large scale non-partisan campaigns for turnout. For (randomized) partisan campaigns, see Gerber et al. (2011), Kendall et al. (2015), Pons (2018), and Braconnier et al. (2017). See Gerber and Green (2017) for a review of field experiments on voter mobilization.

8 Ashworth and Clinton (2006) find no effect of advertising exposure on turnout. Barton et al. (2014) provide evidence from a US local election that canvassing by the candidate is effective in increasing his vote share. Bhatti et al. (2019) question the effectiveness of canvassing outside the US. However, Pons and Liegey (2019) find evidence of an increase in turnout among French immigrants due to visits from political activists.

9 We decided to target a population aged between 18 and 65, because (i) we were only interested in eligible voters, and (ii) people over 65 are unlikely to participate in an online survey. The relevant comparisons follow: aged between 18 and 30, 23% in our sample vs. 22% in Milan; aged between 31 and 45, 46% vs. 43%; aged between 46 and 65, 31% vs. 35%. We also agreed with the “CE &Co” to have a slight over-representation of females, who are more likely to participate in online surveys: females, 59% in our sample vs. 52% in Milan; males, 41% vs. 48%. The neighborhood distribution was similar in the sample and in the general electorate.

10 All surveys are available on the experiment website.

11 The videos and all graphical information were realized by professionals, in collaboration with the opponent campaign, and are available on the experiment website.

12 These gender differences in observables, however, do not represent a threat to the validity of our estimates, as are they balanced across treatment groups within gender strata (Table A.5 in Appendix A).

13 Roberto Basso, spin doctor of Mr. Pisapia’s campaign, in an interview with the newspaper “Europa” (August 21, 2013), defined the attack by Ms. Moratti on Sky TV as the defining moment” of the campaign. He also confirmed that the event was completely unanticipated by Pisapia’s team, as Ms. Moratti was not used to this campaign attacks.

14 The survey questionnaire is available on the experiment website.

15 As in Milan, our sample was not representative of the electorate in the 2015 Cava de’ Tirreni election. The internal validity of the experiment, however, rests on the validity of the randomization protocol.

16 This information was retrieved from the daily diary of the field manager.

17 The distinction between the vote share of the candidate and of the party list is of interest. In fact, according to the electoral rule, mayoral candidates can be supported by one or more party lists, and voters are allowed to cast separate votes for a candidate and for a party list supporting another candidate.

18 To our knowledge, Cantoni and Pons (2021) is the only other canvassing experiment run in Italy. They compare the effect on turnout of canvassing done by paid volunteers vs. canvassing done by local candidates to the city council. Their ground is a 2014 election in a mid-sized town in Northern Italy (38 precincts).

19 Following the protocol proposed by Galasso and Nannicini (2023), to validate our operationalization of the two information treatments ex ante, we randomly assigned the two flyers to 50 university students at Bocconi University who did not know the local political environment in Cava. As the treatments were meant to capture a negative vs. positive message, we asked two questions: 1) whether they perceived the opponent’s message as “negative” against the incumbent, in a scale from 1 to 10; 2) whether they perceived the opponent’s ideological position as “leftist” (as a falsification test), in a scale from 1 to 10. For the 25 students who received the negative message, the average negativity evaluation was 7.280 (s.d. 1.173) and the average ideology evaluation was 5.360 (s.d. 1.319). For the 25 students who received the positive message, these values were 4.720 (s.d. 0.936) and 5.280 (s.d. 1.308), respectively. This test on out-of-sample subjects clearly indicated that the messages were perceived as expected. Neither the negative nor the positive message altered the belief on the ideological position of the sender; this can be seen as a falsification test showing that the issue selection or the word use did not have unintended consequences.

20 These gender differences in observables do not pose a threat to identification, as they do not exhibit systematic differences across treatment arms (see Table C.4), even within gender strata (see Tables C.2 and C.3).

## References

[CR1] Akerlof, G. A., & Kranton, R. E. (2000). Economics and Identity. *Quarterly Journal of Economics,**115*(3), 715–753.

[CR2] Alexander, A. C., Bågenholm, A., & Charron, N. (2020). Are women more likely to throw the rascals out? The mobilizing effect of social service spending on female voters. *Public Choice,**184*, 235–261.

[CR3] Ansolabehere, S., Iyengar, S., Simon, A., & Valentino, N. (1994). Does attack advertising demobilize the electorate? *American Political Science Review,**88*(4), 829–838.

[CR4] Ansolabehere, S., & Iyengar, S. (1995). *Going negative: How political advertisements shrink and polarize the electorate*. The Free Press.

[CR5] Arceneaux, K., & Nickerson, D. W. (2010). Comparing negative and positive campaign messages. *American Politics Research,**38*(1), 54–83.

[CR6] Ashworth, S., & Clinton, J. D. (2006). Does advertising exposure affect turnout? *Quarterly Journal of Political Science,**2*(1), 27–41.

[CR7] Bagues, M., & Esteve-Volart, B. (2012). Are women pawns in the political game? Evidence from elections to the Spanish senate. *Journal of Public Economics,**96*, 387–399.

[CR8] Baltrunaite, A., Bello, P., Casarico, A., & Profeta, P. (2014). Gender quotas and the quality of politicians. *Journal of Public Economics,**118*(C), 62–74.

[CR9] Baron-Cohen, S., Knickmeyer, R. C., & Belmonte, M. K. (2005). Sex differences in the brain: Implications for explaining autism. *Science,**310*(4), 819–823. 16272115 10.1126/science.1115455

[CR10] Barton, J., Castillo, M., & Petrie, R. (2014). What persuades voters? A field experiment on political campaigning. *Economic Journal,**124*(574), 293–326.

[CR11] Bertrand, M. (2010). New perspectives on gender. In D. Card & O. Ashenfelter (Eds.), *Handbook of labor economics, vol 4, part B. *Elsevier.

[CR100] Bhatti, Y., Dahlgaard, J. O., Hansen, J. H., & Hansen, K. M. (2019). Is Door-to-Door Canvassing Effective in Europe? Evidence from a Meta-study across Six European Countries. *British Journal of Political Science*, *49*(1), 279–290.

[CR12] Braconnier, C., Dormagen, J.-Y., & Pons, V. (2017). Voter registration costs and disenfranchisement: Experimental evidence from France. *American Political Science Review,**111*, 584–604.

[CR13] Brooks, D. J., & Geer, J. G. (2007). Beyond negativity: The effects of incivility on the electorate. *American Journal of Political Science,**51*(1), 1–16.

[CR14] Cantoni, E., & Pons, V. (2021). Do interactions with candidates increase voter support and participation? Experimental evidence from Italy. *Economics & Politics,**33*(2), 379–402.

[CR15] Cassese, E. C., & Holman, M. R. (2018). Party and gender stereotypes in campaign attacks. *Political Behavior,**40*, 785–807.

[CR16] Cassese, E. C., & Holman, M. R. (2019). Playing the woman card: Ambivalent sexism in the 2016 U.S. presidential race. *Political Psychology,**40*(1), 55–74.

[CR101] Cavalcanti, T. V., & Tavares, J. (2011). Women prefer larger governments: Growth, structural transformation, and government size. *Economic Inquiry*, *49*(1), 155–171.

[CR17] Chattopadhyay, R., & Duflo, E. (2004). Women as policy makers: Evidence from a randomized policy experiment in India. *Econometrica,**72*(5), 1409–1443.

[CR18] Clinton, J. D., & Lapinski, J. S. (2004). Targeted advertising and voter turnout: An experimental study of the 2000 presidential election. *Journal of Politics,**66*(1), 69–96.

[CR19] Croson, R., & Gneezy, U. (2009). Gender differences in preferences. *Journal of Economic Literature,**47*(2), 448–474.

[CR20] Dewan, T., Humphreys, M., & Rubenson, D. (2014). Elements of political persuasion: Content, contact, or cue. *Economic Journal,**124*(574), 257–292.

[CR21] Edlund, L., & Pande, R. (2002). Why have women become left-wing? The political gender gap and the decline in marriage. *Quarterly Journal of Economics,**117*, 917–961.

[CR22] Finkel, S. E., & Geer, J. G. (1998). A spot check: Casting doubt on the demobilizing effect of attack advertising. *American Journal of Political Science,**42*(2), 573–595.

[CR23] Freedman, P., & Goldstein, K. (1999). Measuring media exposure and the effects of negative campaign ads. *American Journal of Political Science,**43*(4), 1189–1208.

[CR24] Fridkin, K. L., & Kenney, P. J. (2011). Variability in citizens’ reactions to different types of negative campaigns. *American Journal of Political Science,**55*(2), 307–325.

[CR102] Funk, P., & Gathmann, C. (2013). How do electoral systems affect fiscal policy? Evidence from state and local governments, 1890 to 2005. *Journal of the European Economic Association*, *11*(5), 1178–1203.

[CR25] Gagliarducci, S., & Paserman, M. D. (2012). Gender interactions within hierarchies: Evidence from the political arena. *Review of Economic Studies,**79*(3), 1021–1052.

[CR26] Galasso, V., & Nannicini, T. (2023). *Doing experiments with politicians*. Mimeo.

[CR27] Galasso, V., Nannicini, T., & Nunnari, S. (2023). Positive spillovers from negative campaigning. *American Journal of Political Science,**67*(1), 5–21. 37035836 10.1111/ajps.12610 PMC10078752

[CR28] Gerber, A. S., Gimpel, J. G., Green, D. P., & Shaw, D. (2011). How large and long-lasting are the persuasive effects of televised campaign ads? Results from a randomized field experiment. *American Political Science Review,**105*, 135–150.

[CR29] Gerber, A. S., & Green, D. P. (2000). The effects of canvassing, telephone calls, and direct mail on voter turnout: A field experiment. *American Political Science Review,**94*, 653–63.

[CR30] Gerber, A. S., Green, D. P., & Shachar, R. (2003). Voting may be habit-forming: Evidence from a randomized field experiment. *American Journal of Political Science,**47*, 540–50.

[CR31] Gerber, A. S., & Green, D. P. (2017). Field Experiments on voter mobilization: An overview of a burgeoning literature. In *Handbook of economic field experiments*, Vol 1, pp. 395–428. Elsevier.

[CR32] Gilligan, C. (1982). *In a different voice: Psychological theory and women’s development*. Harvard University Press.

[CR33] Glaeser, E. L., Ponzetto, G., & Shapiro, J. M. (2005). Strategic extremism: Why republicans and democrats divide on religious values. *Quarterly Journal of Economics,**120*(4), 1283–1330.

[CR34] Goldstein, K., & Freedman, P. (2002). Campaign advertising and voter turnout: New evidence for a simulation effect. *Journal of Politics,**64*(3), 721–740.

[CR35] Green, D. P., & Gerber, A. S. (2004). *Get out the vote: How to increase voter turnout*. Brookings Institution Press.

[CR36] Herrnson, P. S., Lay, J. C., & Stokes, A. K. (2003). Women running as women: Candidate gender, campaign issues, and voter-targeting strategies. *Journal of Politics,**65*(1), 244–55.

[CR37] Huber, G., Gerber, A., Biggers, D., & Hendry, D. (2022). Can raising the stakes of election outcomes increase participation? Results from a large-scale field experiment in local elections. *British Journal of Political Science,**52*(4), 1635–1650.

[CR38] Kahn, K. F., & Kenney, P. J. (1999). Do negative campaigns mobilize or suppress turnout? Clarifying the relationship between negativity and participation. *American Political Science Review,**93*(4), 877–889.

[CR39] Kahn, K. F., & Kenney, P. J. (2011). Variability in citizens reactions to different types of negative campaigns. *American Journal of Political Science,**55*(2), 307–325.

[CR40] Kendall, C., Nannicini, T., & Trebbi, F. (2015). How do voters respond to information? Evidence from a randomized campaign. *American Economic Review,**105*, 322–353.

[CR41] Krupnikov, Y. (2011). When does negativity demobilize? Tracing the conditional effect of negative campaigning on voter turnout. *American Journal of Political Science,**55*(4), 797–813.

[CR42] Lau, R., Sigelman, L., & Rovner, I. B. (2007). The effects of negative political campaigns: A meta-analytic reassessment. *Journal of Politics,**69*(4), 1176–1209.

[CR43] Muñoz, J., Falcó-Gimeno, A., & Hernández, E. (2020). Unexpected event during survey design: Promise and pitfalls for causal inference. *Political Analysis,**28*(2), 186–206.

[CR44] Nickerson, D. W. (2008). Is voting contagious? Evidence from two field experiments. *American Political Science Review,**102*, 49–57.

[CR103] Niederle, M. (2016). Gender. In J. Kagel & A. E. Roth (Eds.), *Handbook of Experimental Economics*, second edition.

[CR45] Niederle, M., & Vesterlund, L. (2011). Gender and competition. *Annual Review in Economics,**3*, 601–630.

[CR46] Peterson, D. A., & Djupe, P. A. (2005). When primary campaigns go negative: The determinants of campaign negativity. *Political Research Quarterly,**58*(1), 45–54.

[CR47] Pons, V. (2018). Will a five-minute discussion change your mind? A countrywide experiment on voter choice in France. *American Economic Review,**108*(6), 1322–63.

[CR48] Pons, V., & Liegey, G. (2019). Increasing the electoral participation of immigrants: Experimental evidence from France. *Economic Journal,**129*, 481–508.

[CR49] Prakash, V. (1992). Sex roles and advertising preferences. *Journal of Advertising Research,**32*(3), 43–52.

[CR50] Preece, J., & Stoddard, O. (2015). Does the message matter? A field experiment on political party recruitment. *Journal of Experimental Political Science,**2*, 1–10.

[CR53] Stratmann, T. (2006). Contribution limits and the effectiveness of campaign spending. *Public Choice,**129*, 461–474.

[CR54] Teele, D. L., Kalla, J., & Rosenbluth, F. (2018). The ties that double bind: Social roles and women’s underrepresentation in politics. *American Political Science Review,**112*(3), 525–541.

[CR55] Ulbig, S. G., & Funk, C. L. (1999). Conflict avoidance and political participation. *Political Behavior,**21*, 265–82.

[CR56] Wattenberg, M. P., & Brians, C. L. (1999). Negative campaign advertising: Demobilizer or mobilizer? *American Political Science Review,**93*(4), 891–899.

[CR57] Zakharov, A. V. (2009). A model of candidate location with endogenous valence. *Public Choice,**138*, 347–366.

