# Supplementary material for: Persuasion and gender: experimental evidence from two political campaigns
Source: Public Choice. 2024 Aug 5;203(1-2):183–204. doi: 10.1007/s11127-024-01192-y (PMC12213936; doi:10.1007/s11127-024-01192-y)
Supplement: Supplementary file 1 — (pdf 12016 KB) [file 11127_2024_1192_MOESM1_ESM.pdf]

Persuasion and Gender: Experimental Evidence from Two Political Campaigns
Online Appendix

July 17, 2024

Abstract

This Appendix provides additional materials that are also discussed in the paper. In Section A, we report the English translation of our information treatments and additional robustness checks for our survey experiment in Milan. In Section B, we provide information about the sentiment analysis performed for the natural experiment in Milan. In Section C, we report the English translation of our information treatments and robustness checks for our field experiment in Cava de' Tirreni.

Keywords: gender differences, political campaigns, randomized controlled trials.

A Appendix: Survey Experiment in Milan

In the following sections, we report the English translation of our information treatments, and the figures and tables. For complete materials (including original videos realized by professionals, online survey screenshots, etc.) please refer to the experiment website.

A.1 Information Treatments

We exposed individuals in the treatment groups to an entire electoral campaign by the opponent composed of four electoral tools either with a positive (group A) or a negative (group B) tone. All individuals in the two treatment groups were also exposed to the same electoral campaign by the incumbent, again characterized by the same four electoral tools. We now describe our informational treatments.

The first tool of the opponent's randomized campaign was a 100-second video interview to the candidate sitting at his office desk. The second tool was the opponent's main campaign slogan. The third tool was a letter to the voters, which described the opponent's main projects for the future of Milan or charged the incumbent for her mistakes while in office. The final tool was a 60-second video ad endorsed by the opponent on relevant issues for the city (transportation, pollution, Expo). Each of these campaign tools addressed the same issues, with the same format and in the same setting, and was proposed in either a positive or a negative tone. The videos and all graphical information were realized by professionals and are available on the experiment website.

For our experiment, we also simulated the campaign of the incumbent, Letizia Moratti, using the same tools of the opponent's campaign, and we then administered this (non-randomized) campaign to all groups during the second and third survey.

A.1.1 Second Survey: Video Interview with the Opponent

The 100-second videos featured a (single question) interview with the opponent, Giuliano Pisapia, sitting at his desk in his lawyer office. In both the positive and negative version of the video, the opponent addressed four issues: (i) public transportation, (ii) use of bikes, (iii) restrictions to use of the car, and (iv) green areas and parks. After an initial question asked by the same female voice, the candidate response lasted the entire length of the videos. Both videos showed the opponent wearing a white shirt and a tie at his desk,

with a large bookshelf behind him for fifty percent of the time, as they were recorded on the same occasion. For the remaining time, his background voice was accompanied by imagines of traffic, bikes, public transportation, and parks in Milan.

The video with the positive tone—focused on the candidate's proposals on the above topics—ran under the header "my ideas for Milan." The video with the negative tone—focused on the incumbent's main missteps on the same topics during her tenure in office—ran instead under the header "Moratti's mistakes." After each video, a question measured the impact reaction of the respondents to the message ("Do you agree with what the candidate says in the video?").

Positive Toned Video

The video interview with the opponent characterized by a positive tone is available online at the experiment website. The English translation of the text reads as follows.

Interviewer: "How does Giuliano Pisapia plan to solve these problems and increase the quality of life of Milan's citizens?"

Opponent: "I have various ideas. We need to make public transportation an actual alternative to private transport, in particular to cars. We should give everyone the possibility to get around using bicycles. This can be done by extending the bike sharing service, which cannot be limited only to the city center, but should be available also in suburbs. We need to give people the possibility to use bikes as means of transport across the whole city, in the center and in the suburbs. We need new proposals to reduce traffic throughout the city and eliminate it from the city center. I believe that a congestion charge that makes everyone pay a small amount would enhance citizens' well-being. Plus, it would reduce the use of private cars alleviating traffic and pollution. In addition, the revenues from this charge – which needs to be paid by everyone but that will not greatly affect people's budgets – should be invested in public transport. This is the only way to solve problems such as traffic and pollution. Milan should have once again many green spaces and parks; this would greatly benefit not only children, but also adults and elderly. These green spaces can make a positive difference in the lives of our citizens."

Negative Toned Video

The video interview with the opponent characterized by a negative tone is available online at the experiment website. The English translation of the text reads as follows.

Interviewer: “What are the main mistakes made by the Moratti administration in the past five years?”

Opponent: “The mayor proposed to raise the fare for public transportation even though, as citizens know very well, this has been totally inefficient. In these years public transport has been increasingly more off schedule and citizens have had to wait more for buses and trolley cars than in the past. Plus, the speed of transportation has declined continuously. In fact, Milan now ranks 20th in Europe for speed of public transportation with an average speed of 13.5 Km/hr, well below the European average of 20 Km/hr. The Ecopass system is a complete failure; in fact, the councillor who proposed it has been fired. It failed in every respect since it did not reduce traffic (except marginally in the center) and it did not improve the quality of the air we breathe. In Milan, the European critical level of Particulate Matters in the air has been crossed in 35 out of the first 38 days of the year, reaching the European annual limit. This proves that nothing has been done to alleviate traffic and to improve the quality of the air we breathe. Letizia Moratti not only didn’t do anything effective to solve problems such as traffic and pollution, she also did not do anything to create more green spaces in Milan. The worst part is that she wasted a huge present that Maestro Abbado made to our city: 90,000 trees that certainly would have helped make Milan a greener city.”

A.1.2 Second Survey: Video Interview with the Incumbent

The video (available on the experiment website) runs under the header “we want to complete our good work” and broadcasts a public speech by Letizia Moratti, as mayor of Milan, launching her electoral campaign in Piazza San Babila (city center). Surrounded by supporters holding flags and balloons, she promises to complete the projects that were started during her first mandate, with new subway lines being a top priority. The text of the video interview with the incumbent is the following.

“In all these years the center-right administration has always governed well. I have found balanced budgets thanks to mayors Albertini, Formentini, and all those who preceded me. Thanks to this, I have had the possibility to continue to invest. We have invested 3.9 billion Euros in public infrastructures. This allowed us to extend the subway lines: the number 2 line up to Assago has already been inaugurated and the number 3 up

to Comasina has also already been inaugurated. We have also already put aside—they are already registered in our budgets—all the funds necessary to complete the new subway lines 4 and 5 before the Expo."

A.1.3 Second Survey: Electoral Campaign Slogan by the Opponent

The main electoral campaign slogan was shown in a separate page of the survey in a large font and orange (the opponent's electoral campaign color) and black colors. In the positive tone campaign (group A), the slogan (see the original slogan at figure A1) was "Pisapia for Mayor = Less Traffic & More Green. A Change for Milan is Possible." In the negative tone campaign (group B), the slogan (see the original slogan at figure A2) instead was "5 Years of Moratti = More Traffic & Less Green. A Change for Milan is Possible." Each slogan was followed by a question aimed at measuring the respondent's impact reaction ("in general, how much do you feel you can trust Giuliano Pisapia?").

A.1.4 Second Survey: Electoral Campaign Slogan by the Incumbent

The electoral campaign slogan for Letizia Moratti was: "We are working to make Milan an even better place to live in. Letizia Moratti for Mayor."

A.1.5 Third Survey: Open Letter to the Voters by the Opponent

The third tool of the electoral campaign was a one-page (almost two-hundred words long) letter to the voters, signed by the opponent, Giuliano Pisapia. In the initial part of the letter, which was common to both treatments, the opponent expressed his view that the primary duty of a mayor is to increase the wellbeing of the citizens. Then, both in the letter with the positive and the negative tone, he touched upon four issues: (i) clean air; (ii) work ethics; (iii) public transportation; and (iv) involvement of the citizens.

The letter with the positive tone ran (to group A) under the header "this is my commitment with the city," and ended with a positive plea: "Milan deserves to become once again one of the capitals of Europe." The letter with the negative tone ran (to group B) under the header "Milan does not deserve to be led by Ms. Moratti," and ended with a negative plea: "Milan does not deserve other five years of Moratti administration." After each letter, respondents were asked to evaluate the main message ("abstracting from your

political viewpoint, how much do you agree with the general sense of this letter?”).

Positive Toned Letter

The text of the open letter sent to the eligible voters with a positive tone is the following.

“Can a mayor contribute to the happiness of his citizens? I believe he can. Moreover, I am convinced that the primary duty of those who govern in name of the general interest is to increase the wellbeing of their fellow citizens. Together with over a thousand volunteers of the *Workshops for the City*, I have defined four main goals:

1. I want to breathe with you new air, finally clean.
2. I want to live in a city in which work is considered a source of dignity, freedom, and a fundamental value.
3. I want less cars in the city center, more public transportation, a stop a few meters from everyone’s house also in the suburbs, less traffic, and the possibility to move quickly throughout the city also by using bicycles.
4. I want to support those who dedicate their lives to culture; help it thrive sustaining creativity and free initiatives.

I commit to work for these goals. Milan deserves to become once again one of the capitals of Europe.”

Negative Toned Letter

The text of the open letter sent to the eligible voters with a negative tone is the following.

“Can a mayor contribute to the happiness of his citizens? I believe he can. Moreover, I am convinced that the primary duty of those who govern in name of the general interest is to increase the wellbeing of their fellow citizens. In Mrs. Moratti’s Milan this did not happen.

She was indifferent to the city’s problems and rarely present in the city council; therefore, she has proved to be unfit to serve our city.

1. City council. She only attended 5% of ballots, a record high level of absenteeism.

2. City's neighborhoods. Her indifference to the city's needs is obvious: the suburbs are completely abandoned.

3. Little attention was given to transportation and environment, particulate matters in the air are at the highest level since 2007, and 20% of the city's shops pay protection money to the Mafia.

4. Only now—during the electoral campaign—Letizia Moratti is creating a few bikeways and is spending millions of Euros to disseminate throughout the city huge pictures that portray her surrounded by the citizens of Milan.

Milan does not deserve other five years of Moratti administration. Change in Milan is possible."

A.1.6 Third Survey: Open Letter to the Voters by the Incumbent

The header of the incumbent's open letter is "Milan is the city I love." It describes the philosophy that Mrs. Moratti wished to continue to adopt in her second term: to aim high (as with the Expo), but also to take care of the citizens' everyday needs. The text of the letter is the following.

"Milan is the city I love: it is the city in which I was born and raised. It thought me kindness, discretion, and diligence. In my life I have had to deal with situations in which I had great responsibilities. However, nothing is comparable to the emotions I felt when working for my city as Mayor of Milan. In these five years we have aimed at great accomplishments but at the same time we took care of the small necessities of our citizens' everyday lives.

- We aimed high when competing and winning the contest for Expo 2015, which will make Milan capital of the world.

- We took care of small necessities rising security controls in the city's outskirts, investing in street lighting, creating new kindergarten facilities, reaching out to a larger number of elderly, and planting new trees.

I would like to complete the projects we have started in the past five years and make Milan a better place to live in. I will do this with anyone who wants to give the home of all Milanese people more strength, openness and beauty."

A.1.7 Third Survey: Video Ad Endorsed by the Opponent

The last tool of the electoral campaign consisted of a 60-second video of political advertisement realized by professionals and endorsed by the opponent. Both (positive and negative) videos showed images of Milan (such as traffic situations, public transportation, people walking on the streets, the city center, and parks) recorded on the same occasion. The same (professional) speaker read statements on four issues, while relevant synthetic information appeared on the screen. The issues addressed in both videos were: (i) private versus public interest at the city hall; (ii) links between the Expo organization and the mafia; (iii) the management of public appointments; (iv) the city's urban plan.

The video ad with the positive tone ran under the header "my ideas for Milan." The video ad with the negative tone ran under the header "is Ms. Moratti's Milan also your Milan?" The English translations of the messages read in the two videos are in the Appendix. Both videos ended with a common last slogan of endorsement: "Giuliano Pisapia for Mayor." Also in this case, after each video, respondents were asked to evaluate the message ("overall, how truthful does this electoral message seem to you?").

Positive Toned Video

The video ad endorsed by the opponent and characterized by a positive tone is available online at the experiment website. The English translation of the text reads as follows. "The 15th and 16th of May the citizens of Milan will vote to elect their Mayor. Giuliano Pisapia will challenge the incumbent Mayor. What does he plan to do for Milan? To make Milan closer to the needs of citizens, Giuliano Pisapia believes that public service rather than business interests should be a priority [*video highlight appearing on the screen*: 'Close to Citizens' Needs']. To truly fight organized crime, Giuliano Pisapia proposes an anti-Mafia commission that should oversee the works for Expo [*video highlight*: 'Anti-Mafia Commission']. To increase the city's efficiency, Giuliano Pisapia proposes to reward merit and to boost the skills of public employees and managers [*video highlight*: 'Merit and Competence']. To enhance transparency, Giuliano Pisapia believes that the town planning

bill should be discussed with civil society and in the city council [*video highlight*: 'More Transparency']."

Negative Toned Video

The video ad endorsed by the opponent and characterized by a negative tone is available online at the experiment website. The English translation of the text reads as follows. "[*Video highlight appearing on the screen*: '15/16 of May'], Letizia Moratti runs again for Mayor of Milan. Before choosing who to vote, ask yourself whether the Milan she has in mind is also your Milan. [*Video highlight*: '6th of October 2006'], Letizia Moratti undersells optic fibers. Private investors make a profit of 600%, the city looses 50 million Euros [*video highlight*: '+ 600% to private investors'; '- 50 million Euros to the citizens of Milan']. [*Video highlight*: '12th of March 2009'], Letizia Moratti eliminates the anti-Mafia commission that was supposed to oversee the works for Expo [*video highlight*: "infiltrations of the Mafia"]. [*Video highlight*: '24th of May 2009'], investigation on public appointments, the Court of Auditors condemns Letizia Moratti and asks her to refund the city of Milan [*video highlight*: 'illegal public appointments]. [*Video highlight*: '4th of February 2011'], the town planning bill passed by Letizia Moratti tries to amend the one million Euro infringement of building regulations of her son Gabriele [*video highlight*: 'Bat-House']. Is this your Milan?"

A.1.8 Third Survey: Video Ad Endorsed by the Incumbent

The video ad endorsed by the incumbent is available at: http://youtu.be/F9l7BIexZc8. The English translation of the text reads as follows. The video plays under the header "Letizia Moratti: This is my Milan." "Our Milan says 'YES' to more homes for Italians, 'NO' to gypsy camps. 'YES' to a modern Milan, once again leader in the world, 'NO 'to those who live in the past. 'YES' to more assistance and support for the elderly, 'NO' to an administration that raises taxes. 'YES' to more safety and legality, 'NO' to illegal immigration. 'YES' to more aids for mothers." The last scene pictures Letizia Moratti saying "this is my Milan."

A.2 Figures and Tables

Figure A.1: Positive Slogan

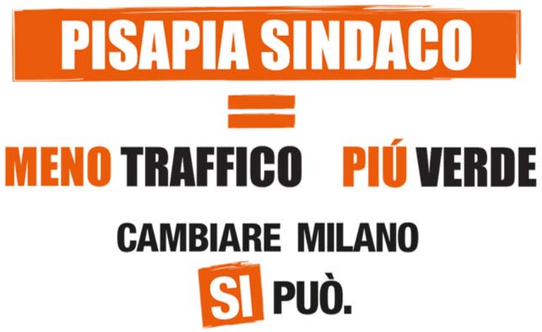

Figure A.2: Negative Slogan

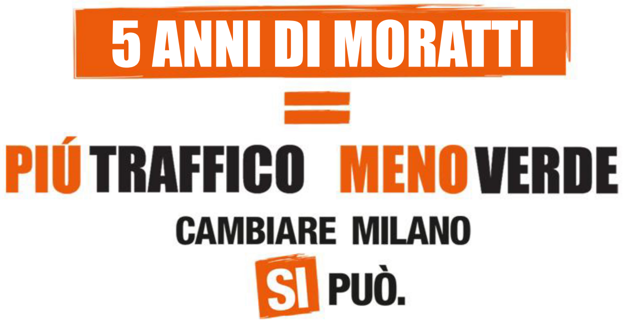

Figure A.3

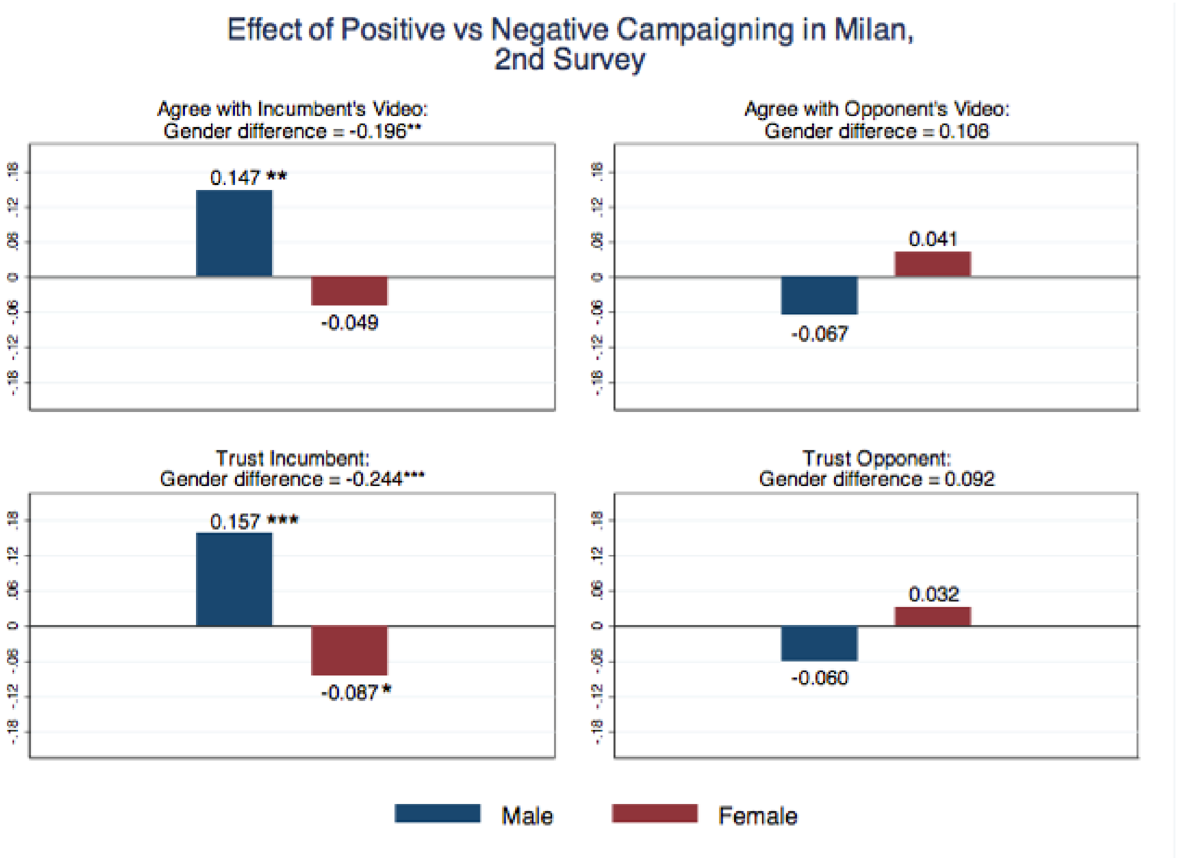

Notes. The "male" estimate captures the treatment effect of positive vs. negative campaign for males: $\alpha_1 - \alpha_2$ in equation 1. The "female" estimate captures the treatment effect of positive vs. negative campaign for females $(\alpha_1 + \beta_1) - (\alpha_2 + \beta_2)$. The "gender difference" estimate captures the differential treatment effect of positive vs. negative campaign between males and females: $\beta_1 - \beta 2$. Significance at the 10% level is represented by *, at the 5% level by **, and at the 1% level by ***.

Figure A.4

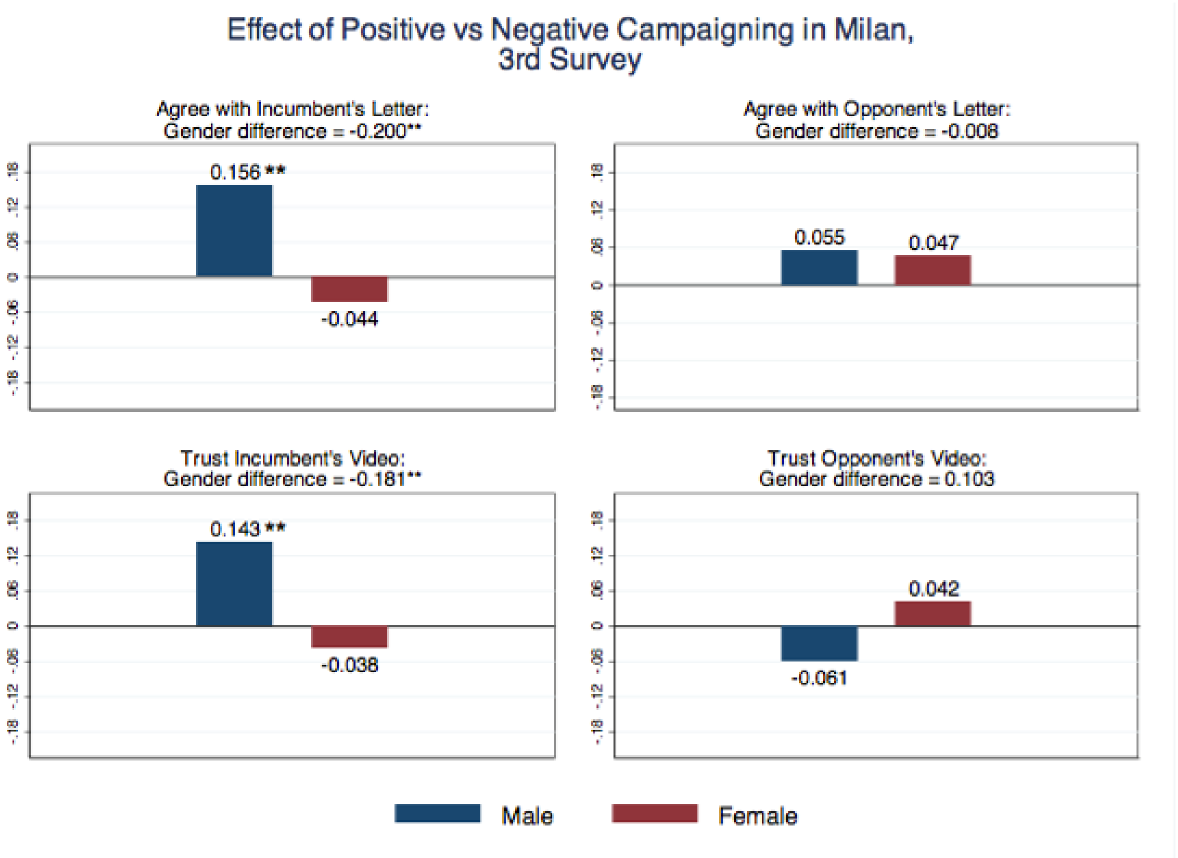

Notes. The "male" estimate captures the treatment effect of positive vs. negative campaign for males: $\alpha_1 - \alpha_2$ in equation 1. The "female" estimate captures the treatment effect of positive vs. negative campaign for females $(\alpha_1 + \beta_1) - (\alpha_2 + \beta_2)$. The "gender difference" estimate captures the differential treatment effect of positive vs. negative campaign between males and females: $\beta_1 - \beta 2$. Significance at the 10% level is represented by *, at the 5% level by **, and at the 1% level by ***.

Table A.1 Descriptive Statistics

	Missing	Female	Young ($\leq$30)	Married	Family with kids	College	Left-wing	Low interest in politics	Did not know mayor
Positive campaign	0.28	0.58	0.23	0.47	0.13	0.44	0.20	0.03	0.03
	(569)	(410)	(410)	(410)	(410)	(410)	(410)	(410)	(410)
Negative campaign	0.24	0.57	0.24	0.47	0.14	0.41	0.17	0.03	0.03
	(568)	(432)	(432)	(432)	(432)	(432)	(432)	(432)	(432)
No campaign	0.25	0.62	0.22	0.51	0.15	0.48	0.17	0.04	0.01
	(399)	(298)	(298)	(298)	(298)	(298)	(298)	(298)	(298)
Total	0.26	0.59	0.23	0.48	0.14	0.44	0.18	0.04	0.03
	(1,536)	(1,140)	(1,140)	(1,140)	(1,140)	(1,140)	(1,140)	(1,140)	(1,140)

Notes. Average values reported; number of observations in parentheses. All variables are dummies. The variable *Left-wing* refers to the (self-declared) ideological position of the voter. *Low interest in politics* captures whether the voter replied to be "hardly" or "not at all" interested in politics. *Did not know mayor* means that the voter was not able to correctly identify the last name of the incumbent mayor.

Table A.2 Covariate Balance Tests

	Missing	Female	Young ($\leq$30)	Married	Family with kids	College	Left-wing	Low interest in politics	Did not know mayor
Positive campaign	0.026	-0.040	0.011	-0.041	-0.014	-0.037	0.021	-0.009	0.021*
	[0.027]	[0.032]	[0.026]	[0.039]	[0.024]	[0.036]	[0.023]	[0.017]	[0.011]
Negative campaign	-0.013	-0.049	0.020	-0.032	-0.011	-0.062	-0.006	-0.014	0.017
	[0.023]	[0.031]	[0.027]	[0.035]	[0.026]	[0.044]	[0.024]	[0.013]	[0.011]
Obs.	1,536	1,140	1,140	1,140	1,140	1,140	1,140	1,140	1,140

Notes. Estimated OLS regression: $Y_i = \alpha_1 POS_i + \alpha_2 NEG_i + \varepsilon_i$. All variables are dummies. The variable *Left-wing* refers to the (self-declared) ideological position of the voter. *Low interest in politics* captures whether the voter replied to be "hardly" or "not at all" interested in politics. *Did not know mayor* means that the voter was not able to correctly identify the last name of the incumbent mayor. Robust standard errors clustered by ZIP code are in brackets. Significance at the 10% level is represented by *, at the 5% level by **, and at the 1% level by ***.

Table A.3 Covariate Balance Tests, Female Voters

	Missing	Young (≤30)	Married	Family with kids	College	Left-wing	Low interest in politics	Did not know mayor
Positive campaign	0.029	-0.028	-0.026	-0.040	-0.037	0.050	-0.012	0.031*
	[0.036]	[0.033]	[0.045]	[0.031]	[0.052]	[0.032]	[0.027]	[0.016]
Negative campaign	-0.009	-0.015	-0.032	-0.004	-0.074	-0.015	-0.014	0.022
	[0.035]	[0.038]	[0.037]	[0.032]	[0.054]	[0.033]	[0.021]	[0.015]
Obs.	907	670	670	670	670	670	670	670

Notes. Estimated OLS regression in the subsample of female voters: $Y_i = \alpha_1 POS_i + \alpha_2 NEG_i + \varepsilon_i$. All variables are dummies. The variable *Left-wing* refers to the (self-declared) ideological position of the voter. *Low interest in politics* captures whether the voter replied to be "hardly" or "not at all" interested in politics. *Did not know mayor* means that the voter was not able to correctly identify the last name of the incumbent mayor. Robust standard errors are in brackets. Significance at the 10% level is represented by *, at the 5% level by **, and at the 1% level by ***.

Table A.4 Covariate Balance Tests, Male Voters

	Missing	Young (≤30)	Married	Family with kids	College	Leftist	Low interest in politics	Did not know mayor
Positive campaign	0.023	0.067	-0.056	0.027	-0.036	-0.017	-0.003	0.006
	[0.049]	[0.043]	[0.067]	[0.038]	[0.049]	[0.041]	[0.018]	[0.018]
Negative campaign	-0.019	0.069	-0.026	-0.016	-0.044	0.012	-0.010	0.009
	[0.047]	[0.050]	[0.061]	[0.039]	[0.065]	[0.044]	[0.015]	[0.019]
Obs.	629	470	470	470	470	470	470	470

Notes. Estimated OLS regression in the subsample of male voters: $Y_i = \alpha_1 POS_i + \alpha_2 NEG_i + \varepsilon_i$. All variables are dummies. The variable *Left-wing* refers to the (self-declared) ideological position of the voter. *Low interest in politics* captures whether the voter replied to be "hardly" or "not at all" interested in politics. *Did not know mayor* means that the voter was not able to correctly identify the last name of the incumbent mayor. Robust standard errors are in brackets. Significance at the 10% level is represented by *, at the 5% level by **, and at the 1% level by ***.

Table A.5 Covariate Balance Tests by Gender

	Missing	Young (≤30)	Married	Family with kids	College	Left-wing	Low interest in politics	Did not know mayor
Positive campaign	0.023	0.067	-0.056	0.027	-0.036	-0.017	-0.003	0.006
	[0.049]	[0.043]	[0.067]	[0.038]	[0.049]	[0.041]	[0.018]	[0.018]
Negative campaign	-0.019	0.069	-0.026	-0.016	-0.044	0.012	-0.010	0.009
	[0.047]	[0.050]	[0.061]	[0.039]	[0.065]	[0.044]	[0.015]	[0.019]
Positive × Female	0.06	-0.095*	0.031	-0.068	-0.001	0.067	-0.009	0.026
	[0.066]	[0.053]	[0.076]	[0.049]	[0.074]	[0.058]	[0.033]	[0.026]
Negative × Female	0.10	-0.084	-0.006	0.012	-0.030	-0.027	-0.003	0.012
	[0.067]	[0.068]	[0.066]	[0.050]	[0.082]	[0.060]	[0.028]	[0.026]
Female	0.002	0.009	0.061	0.038	0.026	0.039	0.028	-0.007
	[0.052]	[0.048]	[0.053]	[0.034]	[0.062]	[0.039]	[0.027]	[0.017]
Obs.	1,536	1,140	1,140	1,140	1,140	1,140	1,140	1,140

Notes. Estimated OLS regression: $Y_i = \alpha_1 POS_i + \alpha_2 NEG_i + \beta_1 POS_i \times FEMALE_i + \beta_2 NEG_i \times FEMALE_i + \delta FEMALE_i + \varepsilon_i$. Robust standard errors are in brackets. Significance at the 10% level is represented by *, at the 5% level by **, and at the 1% level by ***.

Table A.6 Instantaneous Effects of Campaign Tools in Milan

	Panel A. 2nd Survey			
	Agree with opponent's video	Agree with incumbent's video	Trust opponent	Trust incumbent
Positive campaign (α_1)	-0.067	0.147**	-0.060	0.157***
	[0.051]	[0.060]	[0.049]	[0.056]
Positive campaign × Female (β_1)	0.108	-0.196**	0.092	-0.244***
	[0.070]	[0.078]	[0.072]	[0.065]
Female	-0.013	0.076	-0.024	0.088*
	[0.060]	[0.051]	[0.055]	[0.045]
P-value H1: $\alpha_1 + \beta_1$*=0*	0.374	0.384	0.481	0.088*
Obs.	793	793	793	793

	Panel B. 3rd Survey			
	Agree with opponent's letter	Agree with incumbent's letter	Trust opponent's video	Trust incumbent's video
Positive campaign (α_1)	0.055	0.156**	-0.061	0.143**
	[0.060]	[0.059]	[0.058]	[0.057]
Positive campaign × Female (β_1)	-0.008	-0.200**	0.103	-0.181**
	[0.068]	[0.078]	[0.076]	[0.078]
Female	0.005	0.020	0.016	0.023
	[0.049]	[0.049]	[0.058]	[0.053]
P-value H1: $\alpha_1 + \beta_1$*=0*	0.269	0.380	0.359	0.424
Obs.	762	762	762	762

Notes. Estimated OLS regression in the subsample exposed to any campaign (non-missing values only): $Y_i = \alpha_1 POS_i + \beta_1 POS_i \times FEMALE_i + \delta FEMALE_i + \varepsilon_i$. (H1) Treatment effect of positive vs. negative campaign for females: $\alpha_1 + \beta_1 = 0$. In Panel A, the first two columns refer to questions asked after the video interview with each candidate ("do you agree with what the candidate says in the video?"); last two columns refer to questions asked after the campaign slogan of each candidate ("how much do you feel you can trust the candidate?"). In Panel B, the first two columns refer to questions asked after each candidate's open letter to voters ("do you agree with the general sense of this letter?"); last two columns refer to questions asked after the video ad endorsed by each candidate ("how truthful does this electoral message seem to you?"). Robust standard errors are in brackets. Significance at the 10% level is represented by *, at the 5% level by **, and at the 1% level by ***.

Table A.7 Mean Differences by Gender

	Missing	Young (≤30)	Married	Family with kids	College	Left-wing	Low interest in politics	Did not know mayor
Male	0.252	0.264	0.438	0.128	0.430	0.150	0.021	0.023
Female	0.261	0.206	0.509	0.146	0.446	0.201	0.045	0.030
Difference	-0.009	0.058**	-0.071**	-0.019	-0.16	-0.052**	-0.023**	-0.006
P-value	0.708	0.022	0.019	0.371	0.581	0.023	0.034	0.51
Obs.	1,536	1,140	1,140	1,140	1,140	1,140	1,140	1,140

Notes. Average values reported. All variables are dummies. *P-value* captures the statistical significance of the mean difference by gender.

Table A.8 Potential Channels in Milan

	Panel A. Opponent's vote share				
	Baseline	Young (≤30)	College	Left	Low Interest in Politics
P-value: $\alpha_1 = 0$	0.069*	0.022**	0.041**	0.128	0.079*
P-value: $\alpha_2 = 0$	0.284	0.060*	0.314	0.280	0.318
P-value: $\beta_1 = 0$	0.023**	0.022**	0.023 **	0.039**	0.023**
P-value: $\beta_2 = 0$	0.441	0.453	0.438	0.387	0.434
P-value H1: $\alpha_1 + \beta_1 = 0$	0.154	0.397	0.502	0.213	0.148
P-value H2: $\alpha_2 + \beta_2 = 0$	0.85	0.166	0.854	0.978	0.912
P-value H3: $\alpha_1 - \alpha_2 = 0$	0.435	0.997	0.205	0.668	0.408
P-value H4: $\alpha_1 + \beta_1 - (\alpha_2 + \beta_2) = 0$	0.062*	0.026**	0.282	0.118	0.078*
P-value H5: $\beta_1 - \beta_2 = 0$	0.035**	0.058*	0.036**	0.083*	0.036**
P-value H6: $\alpha_1 + \alpha_2 = 0$	0.132	0.024**	0.107	0.170	0.153
P-value H7: $\alpha_1 + \beta_1 + \alpha_2 + \beta_2 = 0$	0.46	0.829	0.763	0.463	0.426
P-value H8: $\beta_1 + \beta_2 = 0$	0.104	0.106	0.105	0.122	0.103
Obs.	912	912	912	912	912
	**Panel B. Incumbent's vote share**				
	Baseline	Young (≤30)	College	Left	Low Interest in Politics
P-value: $\alpha_1 = 0$	0.025**	0.019**	0.016**	0.052*	0.018**
P-value: $\alpha_2 = 0$	0.112	0.029*	0.061*	0.149	0.109
P-value: $\beta_1 = 0$	0.008***	0.007***	0.009***	0.014**	0.009***
P-value: $\beta_2 = 0$	0.201	0.190	0.192	0.165	0.197
P-value H1: $\alpha_1 + \beta_1 = 0$	0.119	0.365	0.465	0.235	0.139
P-value H2: $\alpha_2 + \beta_2 = 0$	0.982	0.321	0.578	0.886	0.988
P-value H3: $\alpha_1 - \alpha_2 = 0$	0.619	0.937	0.474	0.764	0.581
P-value H4: $\alpha_1 + \beta_1 - (\alpha_2 + \beta_2) = 0$	0.073*	0.062*	0.178	0.198	0.088*
P-value H5: $\beta_1 - \beta_2 = 0$	0.076*	0.092*	0.079*	0.164	0.077*
P-value H6: $\alpha_1 + \alpha_2 = 0$	0.032**	0.011**	0.0199	0.057*	0.028**
P-value H7: $\alpha_1 + \beta_1 + \alpha_2 + \beta_2 = 0$	0.342	0.997	0.893	0.438	0.375
P-value H8: $\beta_1 + \beta_2 = 0$	0.034**	0.030**	0.035**	0.037**	0.034**
Obs.	912	912	912	912	912

Notes. Estimated OLS regression: $Y_i = \alpha_1 POS_i + \alpha_2 NEG_i + \beta_1 POS_i \times FEMALE_i + \beta_2 NEG_i \times FEMALE_i + \delta FEMALE_i + \gamma_1'(x_i \times POS_i) + \gamma_2'(x_i \times NEG_i) + \theta' x_i + \varepsilon_i$, where x_i is a respectively one of the following covariates: *Young*, *College*, *Left-wing*, and *Low interest in politics*. P-values are reported for the following Wald tests: Treatment effect of positive vs. no campaign for males: $\alpha_1 = 0$. Treatment effect of negative vs. no campaign for males: $\alpha_2 = 0$. Differential treatment effect of positive vs. no campaign between males and females: $\beta_1 = 0$. Differential treatment effect of negative vs. no campaign between males and females: $\beta_2 = 0$. (H1) Treatment effect of positive vs. no campaign for females: $\alpha_1 + \beta_1 = 0$. (H2) Treatment effect of negative vs. no campaign for females: $\alpha_2 + \beta_2 = 0$. (H3) Treatment effect of positive vs. negative campaign for males: $\alpha_1 - \alpha_2 = 0$. (H4) Treatment effect of positive vs. negative campaign for females: $(\alpha_1 + \beta_1) - (\alpha_2 + \beta_2) = 0$. (H5) Differential treatment effect of positive vs. negative campaign between males and females: $\beta_1 - \beta_2 = 0$. (H6) Treatment effect of any campaign vs. no campaign for males: $\alpha_1 + \alpha_2 = 0$. (H7) Treatment effect of any campaign vs. no campaign for females: $(\alpha_1 + \beta_1) + (\alpha_2 + \beta_2) = 0$. (H8) Differential treatment effect of any campaign vs. no campaign between males and females: $\beta_1 + \beta_2 = 0$. Significance at the 10% level is represented by *, at the 5% level by **, and at the 1% level by ***.

B Appendix: Unexpected Event Design

Performing a sentiment analysis requires to identify a list of stems (root of a word, or of many words), which are relevant to infer the sentiment towards a candidate. A positive stem is related to an emotion, such as joy or love, or to an expression of political support, such as "vote for". Conversely, a negative stem is related to a pessimistic emotion, or to an expression of political dislike. We also included some emoticons as they are widely used on twitter to express feelings. The complete list – reported at table B.1 – contains 108 stems, of which 54 are coded as positive and 54 as negative.

Table B.1 Sentiment Analysis: Positive and Negative Stems

Positive stem	Meaning	Negative stem	Meaning
:)	smile	:(	sadness
;)	wink	anti	against
accordo	agreement	accus-	blame
ador-	worship	arrog-	arrogant
amat-	loved	bugi-	lie
amo-	love	cattiv-	nasty
avanti	forward	coglion-	asshole
batte-	beat	colp-	guilt
bell-	good	comunist-	communist
ben-	well	contrari-	disagreement
brav-	clever	contro	against
buon-	good	criminal-	criminal
cambia-	change	debol-	weak
capac-	capable	delus-	disappointment
competent-	competent	disastr-	disaster
concerto	concert	error-	mistake
consens-	consensus	estrem-	extremism
content-	glad	fals-	false
corrett-	right	fascist-	fascist
dalla parte	support	furt-	theft
divers-	different	insicur-	insecure

Table B.1 Sentiment Analysis: Positive and Negative Stems (continued)

Positive stem	Meaning	Negative stem	Meaning
eccitat-	excited	insult-	insult
favor-	favorite	ladr-	thief
felic-	happy	idiot-	idiot
fort-	strong	imbecill-	fool
forza	incitement	inadeguat-	inadequate
futuro	future	indegn-	unworthy
gentile	kind	mal-	bad
gioia	joy	merd-	shit
giust-	right	mort-	death
grand-	strong	non vot-	not vote for
grazi-	thank	odio	hate
innova-	innovate	opoli	scandal
intelligent-	intelligent-	passat	past
meglio	best	paur-	fear
miglior-	better	pazz-	mad
moderat-	moderate	peggio	worse
nuov-	new	pena	distress
onest-	honest	pericol-	danger
orgoglio	pride	pessim-	very bad
preferit-	favorite	pover-	poor
pro	pro	problem-	problem
salv-	safety	rabbia	angry
sicur-	security	ridicol-	ridiculous
sogn-	dream	ruba-	steal
sostegno	support	sbagli-	wrong
sosten-	support	scandal-	scandal
speranz-	hope	scem-	fool
stim-	esteem	schif-	disgust
tif-	support	scorrett-	unfair
vinc-	win	stronz-	asshole
vittor-	victory	terror-	terror
vot-	vote for	vecchi-	old
xd	smile	violen-	violence

Table B.2 Effects of Sky TV Show on the Tweets' Tone

	Moratti Negative Dummy	Moratti Negative Index	Pisapia Negative Dummy	Pisapia Negative Index
	Panel A. OLS specifications			
After Sky (α_1)	0.016	-0.023	0.044***	0.134***
	[0.020]	[0.037]	[0.014]	[0.033]
After Sky × Female (β_1)	0.094**	0.045	-0.019	0.085
	[0.039]	[0.075]	[0.026]	[0.068]
Female	-0.035	-0.004	-0.003	-0.111*
	[0.029]	[0.058]	[0.020]	[0.059]
P-value H1: $\alpha_1 + \beta_1 = 0$	0.001***	0.727	0.259	0.000***
	Panel B. RD specifications			
After Sky (α_1)	0.127**	0.168*	0.063*	-0.095
	[0.052]	[0.091]	[0.037]	[0.080]
After Sky × Female (β_1)	0.092**	0.049	-0.023	0.061
	[0.039]	[0.075]	[0.026]	[0.066]
Female	-0.036	-0.005	-0.002	-0.093*
	[0.029]	[0.059]	[0.020]	[0.056]
P-value H1: $\alpha_1 + \beta_1 = 0$	0.000***	0.050**	0.353	0.704
Observations	1,811	1,811	1,811	1,811

Notes. Panel A reports the OLS specifications: $Y_i = \alpha_1 AFTER_i + \beta_1 AFTER_i \times FEMALE_i + \delta FEMALE_i + \varepsilon_i$; where $AFTER_i$ is a dummy equal to one if the voter responded before the Sky TV show was aired, and equal to zero otherwise. Panel B reports the RD specifications: $Y_i = \alpha_1 AFTER_i + \beta_1 AFTER_i \times FEMALE_i + \delta FEMALE_i + f(DISTANCE_i) + \varepsilon_i$; where $f(.)$ is a spline third-order polynomial control function, and $DISTANCE_i$ is the distance from the time of the show measured in minutes. (H1) Treatment effect of positive vs. negative campaign for females: $\alpha_1 + \beta_1 = 0$. Robust standard errors are in brackets. Significance at the 10% level is represented by *, at the 5% level by **, and at the 1% level by ***.

Figure B.1

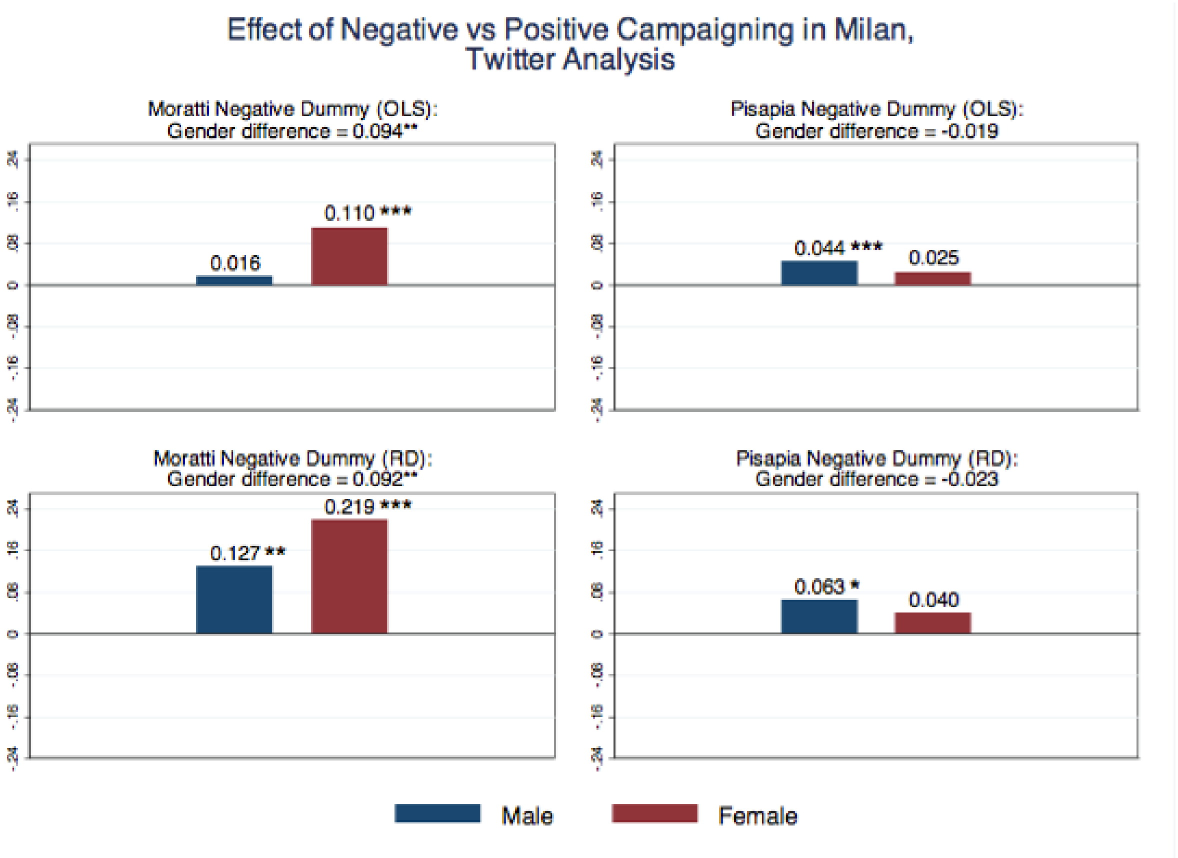

Notes. The "male" estimate captures the treatment effect of positive vs. negative campaign for males: $\alpha_1 - \alpha_2$ in equation 1. The "female" estimate captures the treatment effect of positive vs. negative campaign for females $(\alpha_1 + \beta_1) - (\alpha_2 + \beta_2)$. The "gender difference" estimate captures the differential treatment effect of positive vs. negative campaign between males and females: $\beta_1 - \beta 2$. Significance at the 10% level is represented by *, at the 5% level by **, and at the 1% level by ***.

C Appendix: Field Experiment in Cava de' Tirreni

In the following section, we report the English translation of our information treatments. For complete materials (including original videos realized by professionals, online survey screenshots, etc.) please refer to the experiment website.

C.1 Information Treatments

Our treatments consisted of positive and negative canvassing. During the three weeks prior to the election, a campaign team of volunteers (see figure C1), supporters of Armando Lamberti, knocked on doors of private residences, and buzzed private residences' intercoms (see figure C2), to engage in personal interaction with eligible voters. These personal interactions featured the campaign volunteers soliciting the voters to communicate their ideas about what the new mayor should do for Cava de' Tirreni. These ideas would then be reported to the candidate, Armando Lamberti. Volunteers then took the opportunity to present to these voters Armando Lamberti's ideas, and to distribute electoral material. Electoral material was also left in the mailboxes of other eligible voters, who were not engaged in personal interactions. These electoral materials are at figures C3 to C6. The following sections present respectively the general instructions provided to the volunteers for the canvassing, the script for the initial approach (two options) and the positive and negative messages.

C.1.1 Canvassing Instructions to the Volunteers

General norms to be followed:

- In order to get in touch with the voters and establish a dialogue, it is important to look cheerful, trustful (do not keep your hands in the pocket, do not lean on the wall, no chewing-gums, etc.) and nice. You have to let the voters know that you are not attempting to sell anything, nor you are asking for money, and that you are there to listen to their ideas.
- Avoid assuming aggressive behaviors, even if the person opening the door is clearly aggressive and rude, or if she/he states not to approve Lamberti as a candidate. Just say goodbye and leave.

What to do if the front door of the apartment building is closed:

- If there is a doorman, first convince him/her to let you in the building for the canvassing. It might help starting the canvassing from him/her first. If you succeed, it is likely that he/she will let you in and warn the residents of your arrival. This will likely increase your chances of interviewing a greater number of voters.
- If there is no doorman, you will have to call on the intercom. In order to convince the person to let you in the building you will have to introduce yourselves with one of the introductions you find below.

Once inside the apartment building, how to get personal access to the voters:

- After having entered the building, you have to convince the person to open the door of his/her apartment! Ring the doorbell and when someone answers start introducing yourselves with one of the introductions you find below. The main advantage at this point is that they will see you through the peephole, and they will see your t-shirts.
- If a person, most likely an elderly, decides not to open the door but continues to talk to you from the other side of the door, you can still try to do the canvassing, as described below. You can slid the material under the door.

To enter or not to enter into an apartment:

- You do not need to enter into an apartment for the canvassing. You can give your introductions, listen to their ideas or complaints by remaining on the corridor outside the apartment's door.
- Do not ask to enter into the apartment, people may get frightened. Instead, if you notice that the person is frightened or suspicious, state clearly that you can talk standing at the door.
- If the person invites you to enter, you have two options: (i) If you know the person or he/she looks trustful to you, and you are at least two people, you can enter the apartment and do the canvassing inside; (ii) If you do not feel safe, state that the rules impose you not to enter. If he/she insists, greet him/her and leave.

C.1.2 Canvassing Script I

Good morning/afternoon,

As you can see from our t-shirts, we are young supporters of the candidate mayor Professor Armando Lamberti.

As you might have learned from the newspapers, or as you might have heard from friends of from the streets, Professor Lamberti has promoted an electoral campaign called 'Around the City Listening to Citizens', in order to listen to the ideas and needs of the citizens of the municipality of Cava. We are the volunteers, who 'Listen to Citizens', and we are interviewing people door-to-door.

IF NECESSARY: We know that your time is important and we are not attempting to sell anything, nor we are asking for money. We would like to know what you think the new mayor should do in order to improve the situation in the neighborhood or in your household. Your opinion is fundamental, and Professor Lamberti wants to know which are the priorities to be addressed for the citizens of Cava.

If there is someone else here at home we would also like to talk to him/her in order to collect as many opinions as possible. Once every two or three days, we young supporters meet Professor Lamberti to tell him the citizens' opinions and let him know what really people need.

QUESTION: What is in your opinion the most important issue the new mayor should address? OR ALTERNATIVELY: If you were the mayor, what is the first thing you would do?

C.1.3 Canvassing Script II

Good morning/afternoon,

We are the volunteers, who 'Listen to Citizens'. Have you ever heard of the campaign promoted by the candidate mayor Armando Lamberti to hear the opinions of the citizens of Cava?

As you can see from our t-shirts, we are young supporters of the candidate mayor Professor Armando Lamberti and we would like to know from you what you think of the situation in your neighborhood or in your household, and what the new mayor should do in order to improve the situation.

IF NECESSARY: We know that your time is important and we are not attempting to sell anything, nor we are asking for money. We would like to know what you think the new mayor should do in order to improve the situation in the neighborhood or in your household. Your opinion is fundamental, and professor Lamberti wants to know which are the priorities to be addressed for the citizens of Cava.

If there is someone else here at home we would also like to talk to him/her in order to collect as many opinions as possible. Once every two or three days, we young supporters meet Professor Lamberti to tell him the citizens opinions and let him know what really people need.

QUESTION: What is in your opinion the most important issue the new mayor should address? OR ALTERNATIVELY: If you were the mayor, what is the first thing you would do?

C.1.4 Possible Reactions

There are different tones of possible welcoming, after the opening of the door:

- VERY NEGATIVE WELCOMING (They do not let you speak, they interrupt you, they refuse to open the door or answer that they not have time, or are not interested): Just say thank you, goodbye and leave.

- NEGATIVE WELCOMING (They let you talk, but only in part; they do not open the door and talk from the other side of the door; they say that politicians are all crooks, and that they do not know Lamberti, and vote for XY). You can try one of the following three options: (i) Thank you. We understand that you might not trust politicians, but for us it is still important to know your opinion. What is in your opinion the most important issue the new mayor should address? (ii) Thank you. Even if you do not know Lamberti, for us it is still important to know your opinion. What is in your opinion the most important issue the new mayor should address? (iii) Thank you. Even if you will vote for XY, for us it is still important to know your opinion. What is in your opinion the most important issue the new mayor should address?

- POSITIVE WELCOMING (they let you talk, you arrived successfully to the question in script I or II): After having listened to the answer to your question and having noted it down, you can deliver the following positive or negative message.

C.1.5 Positive Canvassing

How to start:

- Give the person a flyer with the positive message.
- Start from the topic most closely related to the one proposed by the person. Start by stating that that topic is also very important to Professor Lamberti.
- After having talked about the initial topic (the one that is most closely related to the topic proposed by the person), continue BRIEFLY with the two other messages.

Script for the three messages (Only suggestive: you do not need to state everything)

- Dialogue with the citizens: The initiative 'Around the City Listening to Citizens', that brought us here, is just one of the many initiatives Mr Lamberti is planning in order to collect the opinions of the citizens and to dialogue with them, with the goal of taking aware decisions. His dream is that of an inclusive municipality, where every citizen is seen as an important resource. FOR SUB-MUNICIPALITIES (FRAZIONI): This is especially true for the hamlets, which must feel part of the project of creating a unique municipality: Cava. This can be accomplished also through the promotion of public transportation.
- Competency and transparency: in his professional life as a professor of public law and as a member of several regional cabinets, Professor Lamberti has gained a great experience as an administrator, but he has always been very sensitive towards transparency. It is fundamental for him that citizens are aware of his actions and of the decision taken by his cabinet. The main objective is to reduce the burden of bureaucracy and costs, while increasing transparency in the procedures.
- More public services: Professor Lamberti has always been an active promoter of the improvement of the quality and quantity of health care services. He has always

promoted Cava's hospital, and he has also proposed to improve the assistance to citizens by using regional funds that are already available. The project aims at including specialized doctors, a front desk, a nursey, pediatricians, and the launch of the 'Health Center (Casa della salute)', which will be conducting important functions (counseling in support of families, home assistance). The hospital should return to assist acute patients that need hospitalization and those who need emergency interventions, with an emergency service that will remain active 24 hours a day and with wards for cardiology, orthopedics, radiology, intensive care, surgery, pediatrics and the analytical laboratory. FOR SUB-MUNICIPALITIES: this means especially increasing the number of services here in hamlets with health centers and other specialized services of assistance to citizens.

How to finish:

- After having briefly talked about the three themes, ask the person if she/he has any comments. If yes, let the person talk and kindly end the discussion; leave the campaign material, greet him/her and leave.
- If the person interrupts you while you are giving your short presentation, let them him/her talk, try with kindness and BREVITY to talk about all three points. Kindly end the conversation, leave the campaign material, greet him/her and leave.

How to end the conversation if the person wants to continue with the discussion: You can invite him/her to visit Lamberti's committees, which are located in via Verdi. State that the person can meet professor Lamberti and the candidate counselors there.

IMPORTANT: Avoid talking too much if the person is not interested. Better to be brief and avoid getting people bored

C.1.6 Negative Canvassing

How to start:

- Give the person a flyer with the negative message.
- Start from the topic most closely related to the one proposed by the person. Start by stating that over the past five years that issue has not been addressed by Galdi's cabinet.

- After having talked about the initial topic (the one that is most closely related to the topic proposed by the person), continue BRIEFLY with the two other messages.

Script for the three messages (Only suggestive: you do not need to state everything)

- Too much old politics: Galdi's administration has been absent from people's life. Instead of listening to citizens and try to assist their needs, it kept politics distant from people. With Galdi's administration, Cava established a record of cabinets' turnovers: nine turnovers. The main interest of the administration was to keep its 'seats' instead of addressing the citizens' needs. FOR SUB-MUNICIPALITIES: This is especially true for sub-municipalities that joined Cava recently. Lamberti proposes a different style, starting from this initiative of the 'Around the City Listening to Citizens', which brought us here today.

- Too much waste of public money and too many municipal taxes: Galdi's administration pursued a series of wrong public expenditure choices that did not benefit the citizens of Cava. The renovation of the Abbro square with the famous chess-board created many troubles to citizens and waste of public resources. Municipal taxes also increased. For an average household, total municipal taxes increased by 250 Euro per year over the five years of Galdi's administration.

- Too much debt burdening citizens: the purchase of the 'ex-COFIMA' plant by the municipality has raised the level of the municipal debt. The interests on the debt for the purchase of the plant are equal to 1,000 Euro per day. This represents a considerable waste of money, especially because the plant is not yet being used.

How to finish:

- After having briefly talked about the three themes, ask the person if she/he has any comments. If yes, let the person talk and kindly end the discussion; leave the campaign material, greet him/her and leave.

- If the person interrupts you while you are giving your short presentation, let them him/her talk, try with kindness and BREVITY to talk about all three points. Kindly end the conversation, leave the campaign material, greet him/her and leave.

- What to do if the person criticizes your statements about the Galdi's administration? (i) Let him/her talk and interrupt him/her with courtesy; (ii) You can say that the situations you are talking about are complex political and economic issues, and that there can be many different opinions. State that according to you Galdi's administration could have handled certain situations in a better way; (iii) Do not be aggressive and do not attempt to impose your opinion; (iv) Avoid continuing the conversation on Galdi's administration. Kindly greet the person, leave the campaign material and leave.

How to end the conversation if the person wants to continue with the discussion: You can invite him/her to visit Lamberti's committees, which are located in via Verdi. State that the person can meet Professor Lamberti and the candidate counselors there.

IMPORTANT: Avoid talking too much if the person is not interested. Better to be brief and avoid getting people bored.

C.2 Figures and Tables

Figure C.1: Mr Lamberti's Volunteers, Field Experiment in Cava

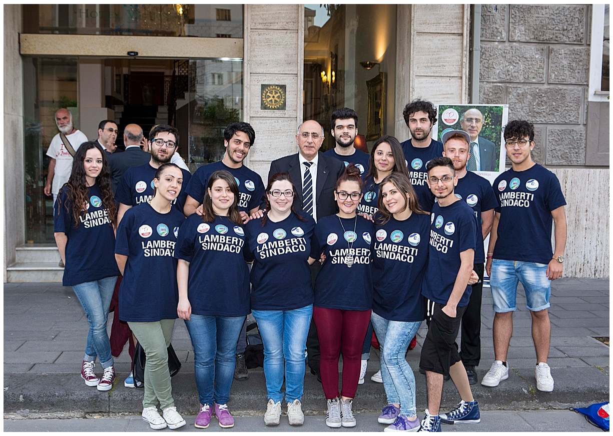

Figure C.2: Mr Lamberti's Volunteers in Action, Field Experiment in Cava

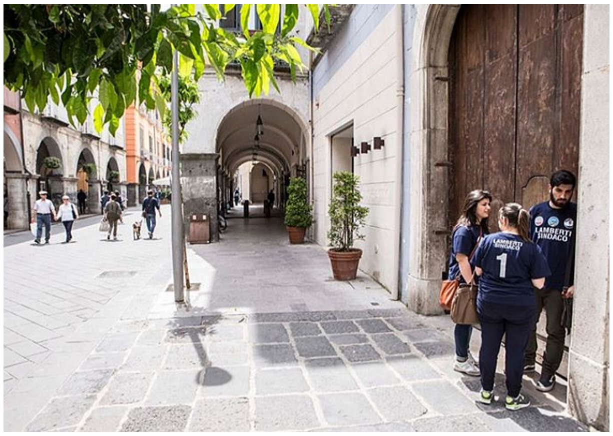

Figure C.3: Flyer with Positive Message, Field Experiment in Cava

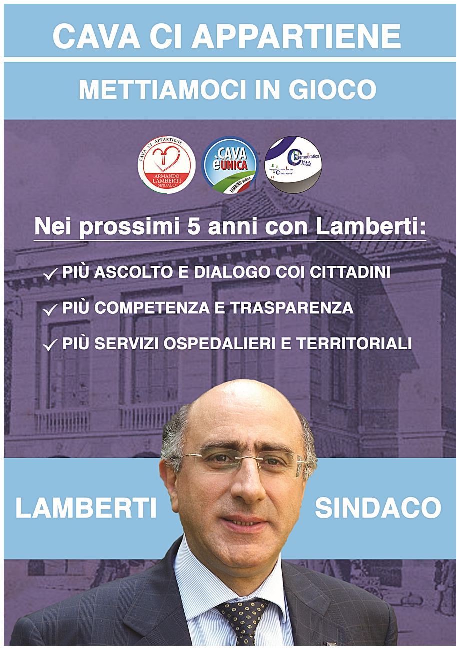

Figure C.4: Flyer with Negative Message, Field Experiment in Cava

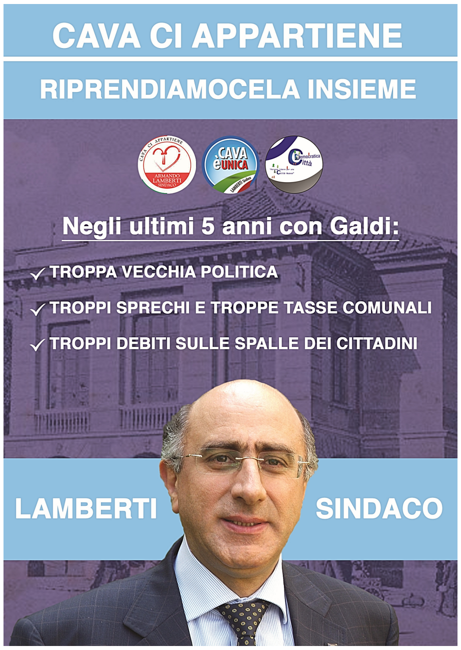

Figure C.5: Hanger with Positive Message, Field Experiment in Cava

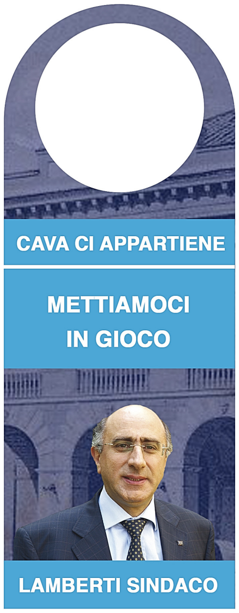

Figure C.6: Hanger with Negative Message, Field Experiment in Cava

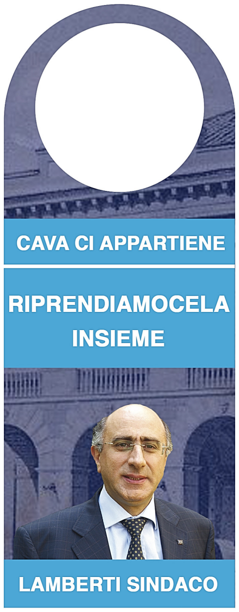

Table C.1 Descriptive Statistics

	Non Canvassed	Female	Young (≤30)	Married	Family with kids	College	Left-wing	Competition	Cooperation
Positive campaign	0.55	0.73	0.06	0.77	0.85	0.18	0.13	0.19	0.68
	279	279	279	261	255	256	279	279	279
Negative campaign	0.51	0.71	0.06	0.75	0.81	0.26	0.13	0.18	0.72
	281	281	281	260	258	262	281	281	281
No campaign	0	0.77	0.07	0.77	0.82	0.23	0.12	0.19	0.69
	297	297	297	272	268	274	297	297	297
Total	0.35	0.74	0.07	0.76	0.83	0.22	0.13	0.19	0.70
	857	857	857	793	781	792	857	857	857

Notes. Average values reported; number of observations in parentheses. All variables are dummies. The variable *Non Canvassed* measures the share of individuals in the initial survey who were not reached by the volunteers, and hence only refers to the treatment groups. *Left-wing* refers to the (self-declared) ideological position of the voter. *Competition* captures whether the voter participated to a professional sport competition or to a contest in his/her life. *Cooperation* means that the voter responded that to cooperate with others is key to have success in life, as opposed to be more competent than the others.

Table C.2 Covariate Balance Tests, Female Voters

	Non Canvassed	Young (≤30)	Married	Family with kids	College	Left-wing	Competition	Cooperation
Positive campaign	0.036	-0.016	0.002	0.044	-0.063*	0.036	-0.029	-0.030
	[0.049]	[0.023]	[0.042]	[0.035]	[0.038]	[0.031]	[0.034]	[0.044]
Negative campaign		-0.030	0.018	0.030	0.016	0.040	-0.036	0.052
		[0.022]	[0.042]	[0.036]	[0.042]	[0.031]	[0.034]	[0.043]
Observations	403	632	587	576	582	632	632	632

Notes. Estimated OLS regression in the subsample of female voters: $Y_i = \alpha_1 POS_i + \alpha_2 NEG_i + \varepsilon_i$. All variables are dummies. The variable *Non Canvassed* measures the share of individuals in the initial survey who were not reached by the volunteers, and hence only refers to the treatment groups. *Left-wing* refers to the (self-declared) ideological position of the voter. *Competition* captures whether the voter participated to a professional sport competition or to a contest in his/her life. *Cooperation* means that the voter responded that to cooperate with others is key to have success in life, as opposed to be more competent than the others. Robust standard errors clustered by ZIP code are in brackets. Significance at the 10% level is represented by *, at the 5% level by **, and at the 1% level by ***.

Table C.3 Covariate Balance Tests, Male Voters

	Non Canvassed	Young (≤30)	Married	Family with kids	College	Left-wing	Competition	Cooperation
Positive campaign	0.052	0.005	-0.030	-0.012	-0.029	-0.071	0.053	0.077
	[0.080]	[0.048]	[0.075]	[0.075]	[0.079]	[0.061]	[0.078]	[0.081]
Negative campaign		0.022	-0.121	-0.091	0.064	-0.069	0.023	0.019
		[0.049]	[0.076]	[0.076]	[0.079]	[0.060]	[0.076]	[0.080]
Observations	157	225	206	205	210	225	225	225

Notes. Estimated OLS regression in the subsample of male voters: $Y_i = \alpha_1 POS_i + \alpha_2 NEG_i + \varepsilon_i$. All variables are dummies. The variable *Non Canvassed* measures the share of individuals in the initial survey who were not reached by the volunteers, and hence only refers to the treatment groups. *Left-wing* refers to the (self-declared) ideological position of the voter. *Competition* captures whether the voter participated to a professional sport competition or to a contest in his/her life. *Cooperation* means that the voter responded that to cooperate with others is key to have success in life, as opposed to be more competent than the others. Robust standard errors clustered by ZIP code are in brackets. Significance at the 10% level is represented by *, at the 5% level by **, and at the 1% level by ***.

Table C.4 Covariate Balance Tests by Gender

	Non Canvassed	Young (≤30)	Married	Family with kids	College	Left-wing	Competition	Cooperation
Positive campaign	0.052	0.005	-0.030	-0.012	-0.029	-0.071	0.053	0.077
	[0.079]	[0.048]	[0.075]	[0.074]	[0.079]	[0.061]	[0.078]	[0.080]
Negative campaign		0.022	-0.121	-0.091	0.064	-0.069	0.023	0.019
		[0.049]	[0.076]	[0.076]	[0.079]	[0.060]	[0.076]	[0.080]
Positive × Female	-0.016	-0.021	0.032	0.056	-0.034	0.107	-0.082	-0.107
	[0.094]	[0.054]	[0.086]	[0.082]	[0.087]	[0.068]	[0.085]	[0.092]
Negative × Female		-0.051	0.139	0.121	-0.048	0.109	-0.059	0.033
		[0.054]	[0.087]	[0.084]	[0.089]	[0.068]	[0.083]	[0.091]
Female	0.133	-0.018	-0.014	0.058	-0.083	-0.095*	-0.133**	0.109
	[0.065]	[0.038]	[0.061]	[0.060]	[0.064]	[0.052]	[0.061]	[0.067]
Observations	560	857	793	781	792	857	857	857

Notes. Estimated OLS regression: $Y_i = \alpha_1 POS_i + \alpha_2 NEG_i + \beta_1 POS_i \times FEMALE_i + \beta_2 NEG_i \times FEMALE_i + \delta FEMALE_i + \varepsilon_i$. All variables are dummies. The variable *Left-wing* refers to the (self-declared) ideological position of the voter. *Competition* captures whether the voter participated to a professional sport competition or to a contest in his/her life. *Cooperation* means that the voter responded that to cooperate with others is key to have success in life, as opposed to be more competent than the others. Robust standard errors are in brackets. Significance at the 10% level is represented by *, at the 5% level by **, and at the 1% level by ***.

Table C.5 Ex-Ante Balancing Tests at the Precinct Level

	Negative Message	Positive Message	Difference (P-Values)
Eligible Voters 2010	25.354	27.576	0.963
	[43.216]	[45.529]	
Eligible Male Voters 2010	14.517	18.073	0.885
	[21.926]	[23.976]	
Eligible Female Voters 2010	10.836	9.503	0.956
	[22.448]	[22.349]	
Turnout 2010	-0.018	-0.011	0.746
	[0.021]	[0.013]	
Center-Right Candidate 2010	0.017	0.029	0.764
	[0.029]	[0.031]	
Center-Left Candidate 2010	-0.013	-0.025	0.657
	[0.030]	[0.031]	
Other Candidates 2010	-0.004	0.000	0.382
	[0.004]	[0.005]	
Center-Right List 2010	0.016	0.024	0.769
	[0.028]	[0.028]	
Center-Left List 2010	-0.011	-0.027	0.516
	[0.029]	[0.029]	
Other Lists 2010	-0.006	0.003	0.109
	[0.005]	[0.006]	
Eligible Voters 2006	33.725	20.781	0.774
	[40.195]	[39.889]	
Eligible Male Voters 2006	16.634	12.690	0.866
	[20.452]	[21.305]	
Eligible Female Voters 2006	17.091	8.091	0.689
	[20.899]	[19.628]	
Turnout 2006	-0.009	-0.011	0.956
	[0.019]	[0.012]	
Center-Right Candidate 2006	-0.028	-0.030	0.258
	[0.022]	[0.016]	
Center-Left Candidate 2006	-0.026	-0.006	0.473
	[0.030]	[0.028]	
Other Candidates 2006	0.054	0.008	0.210
	[0.041]	[0.031]	
Center-Right List 2006	-0.012	-0.000	0.580
	[0.022]	[0.019]	
Center-Left List 2006	-0.028	-0.023	0.812
	[0.024]	[0.026]	
Other Lists 2006	0.041	0.023	0.509
	[0.027]	[0.026]	

Notes. Observations: 55 precincts. OLS coefficients reported; dependent variables in row headings and treatment groups in column headings. *Eligible voters* is the number of voters in the precinct. The other variables are the electoral outcomes in the 2010 and 2006 elections and are expressed in vote shares. Robust standard errors clustered by ZIP code are in brackets. Significance at the 10% level is represented by *, at the 5% level by **, and at the 1% level by ***.

Table C.6 Effects of Campaign Information on Beliefs about Candidates' Campaigns

	Incumbent's ideology	Opponent's ideology	Other's ideology	Incumbent as negative	Opponent as negative	Other as negative
Positive campaign (α_1)	0.273	-0.093	0.344	-0.043	-0.052	-0.098
	[0.230]	[0.303]	[0.350]	[0.110]	[0.118]	[0.091]
Negative campaign (α_2)	0.141	0.120	0.362	0.011	0.267**	-0.137
	[0.220]	[0.286]	[0.344]	[0.109]	[0.117]	[0.085]
Positive campaign × Female (β_1)	-0.064	0.295	-0.279	0.157	0.116	-0.031
	[0.271]	[0.348]	[0.413]	[0.131]	[0.142]	[0.109]
Negative campaign × Female (β_2)	-0.113	0.109	-0.112	0.062	0.030	0.192*
	[0.266]	[0.330]	[0.408]	[0.127]	[0.144]	[0.112]
Female	0.796***	0.579**	1.138***	-0.166*	-0.147	-0.034
	[0.185]	[0.237]	[0.286]	[0.090]	[0.101]	[0.085]
P-value H1: $\alpha_1 + \beta_1 = 0$	0.146	0.245	0.767	0.105	0.415	0.035**
P-value H2: $\alpha_2 + \beta_2 = 0$	0.847	0.164	0.257	0.278	0.001***	0.455
P-value H3: $\alpha_1 - \alpha_2 = 0$	0.568	0.475	0.959	0.622	0.005***	0.606
P-value H4: $\alpha_1 + \beta_1 - (\alpha_2 + \beta_2) = 0$	0.227	0.868	0.414	0.564	0.009***	0.050*
P-value H5: $\beta_1 - \beta_2 = 0$	0.860	0.589	0.690	0.464	0.548	0.026
P-value H6: $\alpha_1 + \alpha_2 = 0$	0.283	0.958	0.239	0.868	0.301	0.139
P-value H7: $\alpha_1 + \beta_1 + \alpha_2 + \beta_2 = 0$	0.346	0.142	0.405	0.107	0.008***	0.529
P-value H8: $\beta_1 + \beta_2 = 0$	0.701	0.491	0.580	0.327	0.558	0.416
Obs.	838	816	829	368	275	303

Notes. Estimated OLS regression: $Y_i = \alpha_1 POS_i + \alpha_2 NEG_i + \beta_1 POS_i \times FEMALE_i + \beta_2 NEG_i \times FEMALE_i + \delta FEMALE_i + \varepsilon_i$. (H1) Treatment effect of positive vs. no campaign for females: $\alpha_1 + \beta_1 = 0$. (H2) Treatment effect of negative vs. no campaign for females: $\alpha_2 + \beta_2 = 0$. (H3) Treatment effect of positive vs. negative campaign for males: $\alpha_1 - \alpha_2 = 0$. (H4) Treatment effect of positive vs. negative campaign for females: $(\alpha_1 + \beta_1) - (\alpha_2 + \beta_2) = 0$. (H5) Differential treatment effect of positive vs. negative campaign between males and females: $\beta_1 - \beta_2 = 0$. (H6) Treatment effect of any campaign vs. no campaign for males: $\alpha_1 + \alpha_2 = 0$. (H7) Treatment effect of any campaign vs. no campaign for females: $(\alpha_1 + \beta_1) + (\alpha_2 + \beta_2) = 0$. (H8) Differential treatment effect of any campaign vs. no campaign between males and females: $\beta_1 + \beta_2 = 0$. Variables description: "Y's ideology" stands for the ideology of Y, measured on a scale from 1 (left) to 5 (right); "Y as negative" is a dummy equal to one if Y's campaign is perceived as negative/aggressive, and zero otherwise. Robust standard errors are in brackets. Significance at the 10% level is represented by *, at the 5% level by **, and at the 1% level by ***.

Table C.7 Mean Differences by Gender

	Non Canvassed	Young (≤30)	Married	Family with kids	College	Left-wing	Competition	Cooperation
Male	0.307	0.098	0.728	0.741	0.305	0.142	0.32	0.636
Female	0.361	0.055	0.775	0.861	0.192	0.120	0.141	0.718
Difference	-0.054	0.042**	-0.047	-0.120***	0.112***	0.0219	0.179***	-0.0827**
P-value	0.143	0.028	0.173	0.000	0.001	0.394	0.000	0.020
Obs.	857	857	793	781	792	857	857	857

Notes. Average values reported. All variables are dummies. *P-value* captures the statistical significance of the mean difference by gender. All variables are dummies. The variable *Non Canvassed* measures the share of individuals in the initial survey who were not reached by the volunteers. *Left-wing* refers to the (self-declared) ideological position of the voter. *Competition* captures whether the voter participated to a professional sport competition or to a contest in his/her life. *Cooperation* means that the voter responded that to cooperate with others is key to have success in life, as opposed to be more competent than the others.

Table C.8 Potential Channels in Cava, Canvassed Sample

	Panel A. Opponent's vote share					
	Baseline	Young (≤30)	College	Left	Competition	Cooperation
P-value: $\alpha_1 = 0$	0.316	0.564	0.349	0.342	0.939	0.019**
P-value: $\alpha_2 = 0$	0.061*	0.040**	0.073*	0.134	0.068*	0.409
P-value: $\beta_1 = 0$	0.034**	0.038**	0.018**	0.024**	0.064*	0.039**
P-value: $\beta_2 = 0$	0.327	0.307	0.389	0.396	0.328	0.279
P-value H1: $\alpha_1 + \beta_1 = 0$	0.060*	0.054*	0.036**	0.063*	0.055*	0.773
P-value H2: $\alpha_2 + \beta_2 = 0$	0.345	0.281	0.385	0.561	0.412	0.768
P-value H3: $\alpha_1 - \alpha_2 = 0$	0.015**	0.020**	0.013**	0.034**	0.041**	0.036**
P-value H4: $\alpha_1 + \beta_1 - (\alpha_2 + \beta_2) = 0$	0.372	0.406	0.392	0.288	0.391	0.612
P-value H5: $\beta_1 - \beta_2 = 0$	0.021**	0.021**	0.014**	0.022**	0.032**	0.017**
P-value H6: $\alpha_1 + \alpha_2 = 0$	0.211	0.143	0.380	0.394	0.163	0.676
P-value H7: $\alpha_1 + \beta_1 + \alpha_2 + \beta_2 = 0$	0.060*	0.046**	0.051*	0.103	0.071*	0.940
P-value H8: $\beta_1 + \beta_2 = 0$	0.659	0.717	0.455	0.584	0.799	0.753
Obs.	282	282	271	282	282	282
	Panel B. Incumbent's vote share					
	Baseline	Young (≤30)	College	Left	Competition	Cooperation
P-value: $\alpha_1 = 0$	0.221	0.167	0.366	0.140	0.455	0.886
P-value: $\alpha_2 = 0$	0.007***	0.004***	0.004***	0.004***	0.014**	0.111
P-value: $\beta_1 = 0$	0.796	0.772	0.741	0.971	0.694	0.857
P-value: $\beta_2 = 0$	0.293	0.300	0.287	0.201	0.268	0.272
P-value H1: $\alpha_1 + \beta_1 = 0$	0.014**	0.008***	0.032**	0.01***	0.015**	0.735
P-value H2: $\alpha_2 + \beta_2 = 0$	0.73*	0.034**	0.050**	0.084*	0.128	0.494
P-value H3: $\alpha_1 - \alpha_2 = 0$	0.189	0.177	0.090*	0.223	0.291	0.241
P-value H4: $\alpha_1 + \beta_1 - (\alpha_2 + \beta_2) = 0$	0.618	0.661	0.980	0.603	0.523	0.773
P-value H5: $\beta_1 - \beta_2 = 0$	0.188	0.183	0.163	0.190	0.201	0.186
P-value H6: $\alpha_1 + \alpha_2 = 0$	0.035**	0.020**	0.048**	0.019**	0.094*	0.333
P-value H7: $\alpha_1 + \beta_1 + \alpha_2 + \beta_2 = 0$	0.011**	0.004***	0.014**	0.01***	0.020**	0.537
P-value H8: $\beta_1 + \beta_2 = 0$	0.677	0.693	0.716	0.502	0.755	0.624
Obs.	282	282	271	282	282	282

Notes. Estimated OLS regression: $Y_i = \alpha_1 POS_i + \alpha_2 NEG_i + \beta_1 POS_i \times FEMALE_i + \beta_2 NEG_i \times FEMALE_i + \delta FEMALE_i + \gamma_1'(x_i \times POS_i) + \gamma_2'(x_i \times NEG_i) + \theta' x_i + \varepsilon_i$, where x_i is a respectively one of the following covariates: *Young*, *College*, *Left-wing*, *Family with kids*, *Married*, *Competition* and *Cooperation*. P-values are reported for the following Wald tests: Treatment effect of positive vs. no campaign for males: $\alpha_1 = 0$. Treatment effect of negative vs. no campaign for males: $\alpha_2 = 0$. Differential treatment effect of positive vs. no campaign between males and females: $\beta_1 = 0$. Differential treatment effect of negative vs. no campaign between males and females: $\beta_2 = 0$. (H1) Treatment effect of positive vs. no campaign for females: $\alpha_1 + \beta_1 = 0$. (H2) Treatment effect of negative vs. no campaign for females: $\alpha_2 + \beta_2 = 0$. (H3) Treatment effect of positive vs. negative campaign for males: $\alpha_1 - \alpha_2 = 0$. (H4) Treatment effect of positive vs. negative campaign for females: $(\alpha_1 + \beta_1) - (\alpha_2 + \beta_2) = 0$. (H5) Differential treatment effect of positive vs. negative campaign between males and females: $\beta_1 - \beta_2 = 0$. (H6) Treatment effect of any campaign vs. no campaign for males: $\alpha_1 + \alpha_2 = 0$. (H7) Treatment effect of any campaign vs. no campaign for females: $(\alpha_1 + \beta_1) + (\alpha_2 + \beta_2) = 0$. (H8) Differential treatment effect of any campaign vs. no campaign between males and females: $\beta_1 + \beta_2 = 0$. Significance at the 10% level is represented by *, at the 5% level by **, and at the 1% level by ***.
